# Chemosensory System Decoding: Transcriptome‐Wide Identification and Expression Profiling of Olfactory Genes in *Lytta sifanica*

**DOI:** 10.1002/ece3.72634

**Published:** 2025-12-10

**Authors:** Feng Zhou, Zhuan‐xia Li, Jia‐ni Chen, Xin‐ge Song, Shu‐ning Sun, Yu‐ying Zhang, Li‐yuan Yao, Yu‐qin Wang, Xin‐yu Sun, Li‐xia Wan

**Affiliations:** ^1^ College of Life Science Northwest Normal University Lanzhou China

**Keywords:** antennal transcriptome, blister beetle, chemosensory gene, gene expression analysis, gene identification

## Abstract

The insect olfactory system relies on a sophisticated repertoire of olfactory‐related proteins to enable precise odor detection and subsequent signal transduction. Their expression and regulation are crucial for mediating communication between themselves and environments. *Lytta sifanica* (Coleoptera: Meloidae), a species of significant economic importance, represents a notable subject for olfactory studies, particularly as it is renowned for producing the medically relevant toxin cantharidin. However, the molecular basis of olfactory sensation in the Blister Beetle *Lytta sifanica* has not yet been characterized. In this study, the transcriptomes of adult *L. sifanica* antennae were sequenced and analyzed. A total of 70 chemosensory genes, including 17 odorant binding proteins (OBPs), 5 chemosensory proteins (CSPs), 13 gustatory receptors (GRs), 17 odorant receptors (ORs), 13 ionotropic receptors (IRs) and 5 sensory neuron membrane proteins (SNMPs) were identified based on sequence homology analysis and phylogenetic reconstruction. The expression profiles of all candidate genes were confirmed by RT‐PCR across multiple tissues, including antennae, head, mouthparts, pronotum, foreleg tarsus, abdomen skin, and wings. The results revealed that olfactory‐related protein families were broadly expressed in the tested tissues, though with distinct tissue‐specific patterns among different gene families. Among the identified chemosensory genes, eleven members are predominantly expressed in the antennae (*Lsif_OBP1/2/19d, Lsif_OR2/20/49b, Lsif_IR2a, Lsif_GR7/127, Lsif_CSP1*, and *Lsif_SNMP2/4*). In contrast, twenty‐two members were exclusively expressed in the mouthparts (*Lsif_OBP70/56d2, Lsif_GR12a/21/28a/68a*), foreleg tarsus (*Lsif_OR6/67c, Lsif_GR12,* and *Lsif_OBP2*) and were abundant in the non‐olfactory tissues head (*Lsif_OBP99a, Lsif_OR9a/49b, Lsif_IR25a/56e,* and *Lsif_CSP6*), pronotum (*Lsif_OBP5/C20, Lsif_ORco, Lsif_OR13*, and *Lsif_IR7*), abdomen skin (*Lsif_SNMP5*), suggesting their various functions in the olfactory system of *L. sifanica*. This research offers an extensive resource for investigating the chemoreception mechanism in beetle *L. sifanica*.

## Graphical abstract

*Lytta sifanica* (Coleoptera: Meloidae), a species of significant economic importance, is renowned for its production of the toxic compound cantharidin. In this research, Zhou and colleagues collected specimens of this insect and identified seventy olfactory‐related genes through transcriptomic analysis of adult antennae in *L. sifanica*. Sequence homology analysis and phylogenetic reconstruction were performed to elucidate the family characteristics and evolution of the olfactory‐related protein in this beetle. The expression profiles of all candidate genes across seven distinct adult tissues (antennae, head, mouthparts, pronotum, foreleg tarsus, abdominal integument, and wings) were further analyzed using RT‐PCR.

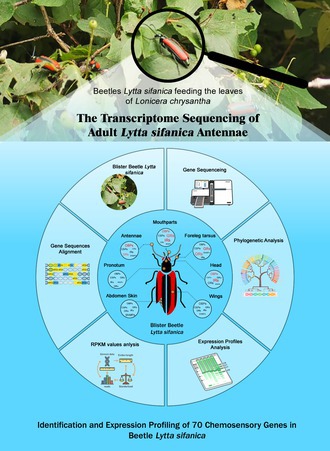

## 1 Introduction

Insects represent the most diverse and widely distributed group of animals on earth. They live in various ecological and changed chemical surroundings, in which insects should properly understand those chemical signals (Yang et al. 2025). These signals, derived from food, sex, predators, pathogens, oviposit, and inhabit provide distinct chemical cues that insects must detect and respond to efficiently. Consequently, their olfactory systems have evolved to be remarkably complex and versatile, with associated functions that are highly developed, tightly regulated, and capable of discerning a broad spectrum of environmental chemical cues (Renou and Anton 2020). For example, the beetle *Brontispa longissima* located its food by smelling the volatile compounds from related plants or animals (Bin et al. 2017). *Colaphellus bowringi* find their mates or predators by identifying the odor through the pheromones released by others (Li et al. 2015). Conversely, *Drosophila melanogaster* with knocked‐out odorant‐binding protein (OBP) genes exhibited significantly reduced attraction to *Morinda citrifolia* ripe fruits. This behavioral deficit was attributed to the flies' impaired response to two key fruit volatiles: hexanoic acid and octanoic acid (Zhan et al. 2021).

Olfactory system of insects is sophisticated, involving a variety of related functional proteins, such as odorant‐binding proteins (OBPs), chemosensory proteins (CSPs), olfactory receptors (ORs), ionotropic receptors (IRs), gustatory receptors (GRs), and sensory neuron membrane proteins (SNMPs) (Gu et al. 2015). Each protein family comprises multiple members that perform distinct functions in insect odor reception. When odorant molecules enter the sensillum lymph, they will first bind to specific odorant‐binding proteins (OBPs). The formed complexes are then transported to odorant receptors (ORs) located on olfactory sensory neurons (OSNs), where the chemical signal is transduced into electrical signals. Finally, the signaling molecules are degraded by specific enzymes to terminate the olfactory response (Ha and Smith 2022). OBPs and CSPs play crucial roles in insect odorant reception by binding, solubilizing, and transporting hydrophobic odorants within antennal sensilla. IRs are involved in the perception of humidity and temperature. GRs are thought to be related to the perception of sugars, bitter‐tasting compounds, non‐volatile compounds, and carbon dioxide. SNMPs are found to play important roles in sensing pheromones (Yang et al. 2024). These molecular components represent conserved elements of insect olfactory systems. Nevertheless, their composition varies significantly across species, with differences in expressions, numbers and sequences of gene families that reflect interspecific changes or specific ecological adaptations. These variations help them to meet their specific needs or habits, which gives important significance to the in‐depth investigation of olfactory genetic variations.

*L. sifanica* (Coleoptera: Meloidae) is a significant agricultural pest that inflicts substantial damage to various host plants, including *Sophora japonica* , willows, and poplars (Pan and Guo‐dong 2018). They are also important predators of locusts and other coleoptera, as a result of their larval predation on locust eggs. Interestingly, the adults secrete a defensive and irritant compound known as (C_10_H_12_O_4_), which is biosynthesized in secretory glands and is characterized by a pungent odor (Aoun et al. 2018). When threatened, beetles release this vesicant agent through reflex bleeding, causing dermal blistering, hence their common designation as “blister beetles”. In recent years, cantharidin has been utilized in the treatment of various diseases, such as tumors and cancer, and shows broad medical application value (Lin et al. 2016). However, the molecular mechanisms by which olfactory genes contribute to defensive responses against predators through cantharidin production, and their possible roles in mediating foraging interactions with plants and other animals, remain poorly understood (Fratini et al. 2022).

Research on the genetic architecture of beetle olfactory systems was pioneered in *Tribolium castaneum* , which served as the foundational model for elucidating olfactory gene function (Trebels et al. 2021). Subsequent investigations have expanded to other economically significant beetle species, notably *Rhynchophorus ferrugineus*, where comprehensive analyses of olfactory‐related genes have been conducted across multiple functional tiers, from molecular characterization to behavioral validation (Huang et al. 2023). Consistent with other insect taxa, beetles have evolved characteristic olfactory genes and formed their families (Guan et al. 2020). Notably, their olfactory genes exhibit partial conservation and functional divergence, which may underlie the diversification of olfactory functions during ecological adaptation (Renou and Anton 2020). In the present study, we focused on the genetic basis of the olfactory system in *L. sifanica*. Among related blister beetles, only a few have had their olfactory genes characterized. For instance, *Hycleus Cichorii* and *Hycleus phaleratus* have been fully sequenced and their antennal olfactory‐related genes have been analyzed using genomic and transcriptomic approaches (Wu, Liu, and Chen 2018). However, the gene identification in other blister beetles remains limited. Currently, genomic investigations of olfactory genes in *L. sifanica* remain limited, with only a small fraction of olfactory gene members have been characterized by Illumina sequencing (Wu, Li, and Chen 2018).

In this study, we investigate the olfactory genetic system of *L. sifanica* using transcriptome sequencing technology. Overall, seventy olfactory‐related genes in *L. sifanica* were identified. Through comparative analysis of sequence data and phylogenetic trees across olfactory gene families, this study has determined the divergence time nodes of olfactory subfamilies in Coleoptera, while functional differentiation pathways were inferred via identification of orthologous and paralogous genes. Their expression patterns were further examined across multiple tissues through the integration of RT‐PCR and FPKM‐based analyses. This work is the first to identify the chemosensory gene families in the beetle species *L. sifanica* and to analyze their related expression profiles, providing a foundation for future investigations into the olfactory mechanisms of this species.

## 2 Materials and Methods

### 2.1 Insects

**FIGURE 1:**
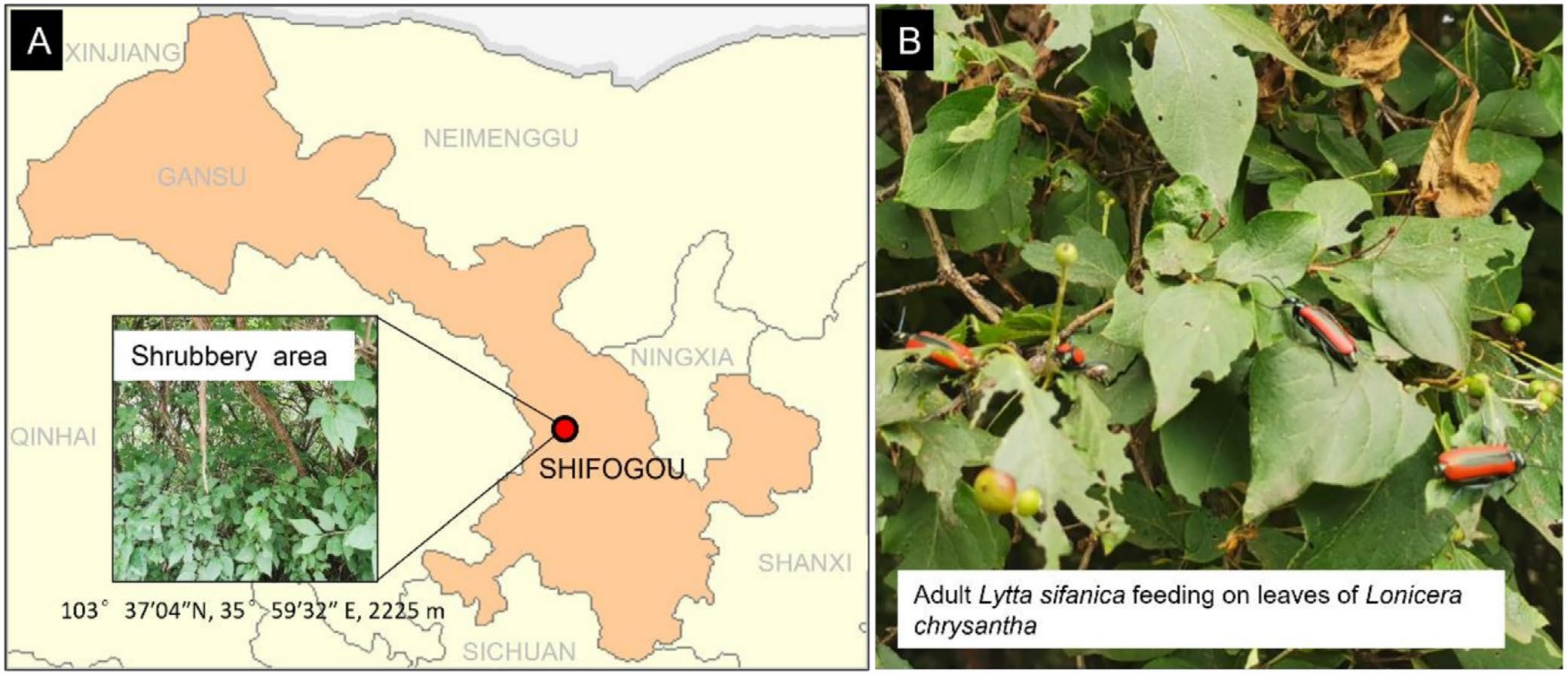
The location of sample collection. (A) Collection site information and habitat. (B) Photos of *L. sifanica* feeding plant *Lonicera chrysantha* .

The antennae of *L. sifanica* were used as the biological materials in this study. Adult *L. sifanica* specimens were collected from Shifogou (103°37′04″ N, 35°9′32″ E), Lanzhou city, GanSu province, China, in early May 2023 (Figure 1A). At the time of collection, the local temperature was approximately 22°C, the relative humidity was 30%, and the average altitude was 2225 m. The beetles observed feeding on the leaves of *Lonicera chrysantha* were shown in the picture (Figure 1B). A total of 105 adult *L. sifanica* were collected subsequently identified based on their morphological characteristics. The identified specimens were then transferred to sterile plastic containers supplied with fresh leaves of *L. chrysantha* for feeding.

### 2.2 Sample Processing and RNA Extraction

In the laboratory, the insects were immediately placed in the freezer (−80°C) for about 5 min for immobilization. Sixty adults (mixed sexes) of comparable size and vigor were selected for antenna dissection using sterile surgical scissors, while the remaining carcasses were preserved for tissue‐specific expression analyses. All antennae tissues were immediately frozen in liquid nitrogen and subsequently stored at −80°C for subsequent experiments. The antennae samples of beetle were further divided into three groups (20 beetle antennae each group) for total RNA isolation. Total RNA was extracted using TRIzol reagent (Life Technologies) according to the manufacturer's instructions. The RNA samples were treated with RNase‐free DNase I (Life Technologies) to eliminate contaminating genomic DNA. Subsequently, RNA integrity and quality for each group were assessed using 1% agarose gel electrophoresis and an Agilent 2100 Bioanalyzer (Agilent Technologies). Only high‐quality RNA samples were used for subsequent library preparation.

### 2.3 cDNA Library Construction and Transcriptome Sequencing

For each sample, 10 μg of total RNA was used for mRNA isolation using oligo(dT) magnetic beads. The cDNA libraries were constructed using the NEBNext mRNA Library Prep Reagent Set (NEB, Ipswich, MA, USA) according to the manufacturer's protocol. The libraries were sequenced on the Illumina HiSeq 2000 (Illumina, San Diego, CA, USA) by BioMarker company (Beijing, China). Libraries were constructed with 1.5 μg purified RNA using a TruSeq RNA Sample Preparation Kit (Illumina, San Diego, CA, United States) following the manufacturer's instructions (Huang et al. 2016). Raw reads were processed to remove adapter sequences, low‐quality reads, and reads containing ambiguous bases, resulting in high‐quality clean reads for downstream analyses.

### 2.4 Transcriptome Data Assembly

High‐quality clean reads obtained from Illumina sequencing were *de novo* assembled using Trinity software (Grabherr et al. 2011) (Version: r2013‐11‐10) with the following parameters: min contig_length (the minimum length of assembled contigs): 200; group_pairs_distance (insert size): 500 bp. All other parameters were set to their default values. The longest transcript within each cluster was defined as a unigene. During the assembly process, reads were fragmented into smaller units known as k‐mers, which served as seeds for contig extension. Contigs were subsequently assembled into components based on sequence overlaps. The open reading frames (ORFs) of the assembled unigenes were predicted using the NCBI ORF Finder tool (http://www.ncbi.nlm.nih.gov/gorf/gorf.html) (Langmead and Salzberg 2012).

### 2.5 Functional Annotation

Unigene sequences were comprehensively annotated by performing similarity searches against multiple public databases, including the NCBI non‐redundant protein (Nr), Swiss‐Prot, Gene Ontology (GO), Clusters of Orthologous Groups (COG), euKaryotic Orthologous Groups (KOG), evolutionary genealogy of genes: Non‐supervised Orthologous Groups (eggNOG), and Kyoto Encyclopedia of Genes and Genomes (KEGG) databases. BLASTx searches were carried out against the Nr protein database with an E‐value cutoff of 1e−5 to obtain preliminary functional annotations. The resulting BLAST outputs were subsequently processed using the Blast2GO pipeline for GO annotation. The longest ORF for each unigene was predicted using the NCBI ORF Finder tool (http://www.ncbi.nlm.nih.gov/gorf/gorf.html). Amino acid sequences were predicted using TransDecoder, and and protein domains were identified by searching these sequences against the Pfam database using HMMER (Nastou et al. 2016). The E‐value thresholds were set at ≤ 1e−5 for BLAST and ≤ 1e−10 for HMMER. KO terms and metabolic pathway annotations were assigned using KOBAS 2.0 (Kanehisa et al. 2008).

### 2.6 Genes Identification

To systematically identify chemosensory‐related genes in *L. sifanica*, a total of 1186 chemosensory proteins sequences from other beetles species were downloaded from NCBI database as reference sequences. Reference species included *Agrilus planipennis* (14), *Dastarcus helpophoroides* (37), *Dendroctonus adjunctus* (32), *Dendroctonus ponderosae* (68), *Dendroctonus valens* (46), *H. cichorii* (374), *H. phaleratus* (286), *Ips typographus* (41), *Monochamus alternatus* (55), and *T. castaneum* (233) (Table S2). Sequence similarity searches were conducted between these reference sequences and the *L. sifanica* transcriptome database using local BLASTx. The candidate sequences with highly similar to the bait chemosensory proteins were further searched against the NCBI Nr database to determine their homology. An addition, protein family analysis was performed on all identified sequences based on the characteristic patterns of OBPs, CSPs, ORs, GRs, IRs, and SNMPs, respectively. Finally, all identified sequences were integrated using BLASTx and BLASTn software and produced a non‐redundant gene set for manual examination (https://blast.ncbi.nlm.nih.gov/Blast.cgi). Signaling peptides for odor‐binding proteins and chemosensory proteins were predicted using SignalP 6.0—DTU Health Tech (Dippel et al. 2014). The expression levels of different olfactory genes in the antennae of *L. sifanica* were evaluated using FPKM values (Qiu et al. 2020). All identified chemosensory proteins were used for motif identification and analysis. Motif identification and analysis were performed using MEME (Version 5.1.1) on the online server (https://meme‐suite.org/meme/) with the following parameters: minimum width = 6, maximum width = 10, and maximum number of motifs = 8.

### 2.7 Phylogenetic Analysis

To further explore the evolutionary patterns of chemosensory proteins in *L. sifanica*, we constructed a phylogenetic tree using olfactory‐related amino acid sequences identified from *L. sifanica* and other beetle species. Amino acid sequences of identified OBPs, CSPs, ORs, IRs, GRs, and SNMPs from *L. sifanica* were aligned with homologous olfactory‐related proteins from other Coleoptera species, including *H. cichorii*, *H. phaleratus* , *D. ponderosae* , and *T. castaneum* (Table S3). The selection of outgroups in this study was based on phylogenetic proximity (e.g., coleopteran species) and supporting evidence from published phylogenies (particularly those focusing on olfactory gene evolution). First, multiple sequence alignments were performed for all members of each chemosensory protein family using ClustalX 2.0 (Larkin et al. 2007). Aligned sequences were used to calculate the best substitution model for each gene family in ProtTest 3.449 (Tamura et al. 2013). The matrices of sequence alignments were further used to estimate the best‐fitting substitution model using MEGA v7.0 (Kumar et al. 2016). Consensus rooted trees were reconstructed via maximum‐likelihood (ML) analysis with 1000 bootstrap replicates using MEGA v7.0, and the resulting tree files were imported into the iTOL online server (https://itol.embl.de/) for further optimization. Phylogenetic trees were colored and arranged by using Fig Tree with the LG or VT substitution model for the chemosensory proteins and GAMMA correction (Version: 1.4.2).

### 2.8 RT‐PCR

To characterize the tissue‐specific expression profiles of identified chemosensory proteins in *L. sifanica*, we dissected the head, foreleg tarsus, pronotum, abdomen skin, wings, mouthparts, and antennae of *L. sifanica*. Total RNA was isolated from each tissue type using TRIzol Reagent (Invitrogen) following standardized protocols. Total RNA was isolated from each tissue type using TRIzol Reagent (Invitrogen) according to the manufacturer's instructions, and RNA purity and concentration were quantified using a Nanodrop 2000 spectrophotometer (Thermo Fisher Scientific). Genomic DNA contamination was removed by treating 1 μg of total RNA with DNase I (Takara), and first‐strand cDNA was subsequently synthesized using Oligo(dT)15 Primer (Promega) and M‐MLV Reverse Transcriptase (Invitrogen) following the manufacturer's protocol. The tissue‐specific expression profiles of OBPs, ORs, CSPs, SNMPs, GRs, and IRs were initially evaluated via RT‐PCR to confirm their expression patterns across different tissues. NADH was evaluated and used as the most stable reference gene for gene expression profiling analysis (Table S5). RT‐PCR was performed using 2 × F8 FastLong PCR MasterMix (Aidlab) with gene‐specific primers (Table S5) under the following conditions: initial denaturation at 95°C for 5 min, followed by 35 cycles of 95°C for 30 s, annealing at 55°C–60°C (depending on primers) for 30 s, and extension at 72°C for 45 s, with a final extension at 72°C for 10 min. RT‐PCR products were separated using 1% agarose gel electrophoresis, stained with ethidium bromide (EB), and visualized using a GelDoc XR+ imaging system (Bio‐Rad).

### 2.9 Statistical Analysis

All experimental data were analyzed using InStat statistical software (GraphPad Inc., San Diego). Tissue‐specific expression differences of chemosensory genes were evaluated through one‐way analysis of variance (ANOVA) with a nested design, followed by Duncan's multiple range test for post hoc comparisons (significance threshold α = 0.05). Data are presented as mean ± standard error (SE) of three biological replicates.

## 3 Results

### 3.1 Transcriptome Sequencing and Assembly

In total, approximately 18.12 Gb raw sequencing data were generated from the antennae of *L. sifanica*. After removing adapter sequences and low‐quality reads, the data of each sample had reached 5.73 Gb. The Q30 percentage of all clean data exceeded 90.36%, and the average GC content was 37.91%. De novo assembly of pooled clean reads from three biological replicates produced, a comprehensive transcriptome comprising 66,945 transcripts (average length = 1572 bp). A total of 35,136 unigenes were identified, with an N50 value of 1629 bp. Length distribution analysis indicated that 8214 unigenes, which accounted for 23% of all unigenes, were longer than 1 kb (Figure S1). The clean reads were deposited in the Sequence Read Archive (SRA) database of the National Center for Biotechnology Information (NCBI) under the BioProject accession number: PRJNA1221373.

### 3.2 Functional Annotation and Species Homology Analysis

A total of 17,094 unigenes (48.7% of all unigenes) were annotated using multiple databases including COG, GO, KEGG, KOG, Pfam, Swissprot, TrEMBL, eggNOG, NR, and the numbers (%) were 3984 (23%), 14,079 (82%), 12,053 (70%), 9942 (58%), 11,277 (65%), 7544 (44%), 15,914 (93%), 12,526 (73%), and 15,801 (92%) respectively (Figure S2A). NR database analysis (BLASTx, *E*‐value ≤ 1e‐5) revealed the closest homology with *Tribolium castaneum* (35.4% of annotated unigenes), followed by *Asbolus verrucosus* (21.2%) and *Bombus terrestris* (5.4%). The number (and proportion) of genes annotated to other species in all annotated genes is shown in (Table S4). Gene ontology (GO) analysis was performed to classify the annotated unigenes into three main functional categories: biological process (BP), molecular function (MF), and cellular component (CC). In the BP category, cellular process was the most highly represented subcategory, followed by “metabolic” and “biological regulation” processes. In the MF category, the subcategories “binding” and “catalytic activity” were the most represented. In the CC category, the most highly represented subcategories were cellular anatomical entity, protein‐containing complex, and intracellular part (Figure S2B).

### 3.3 Candidate Odorant Binding Proteins (OBPs) Analysis

**FIGURE 2:**
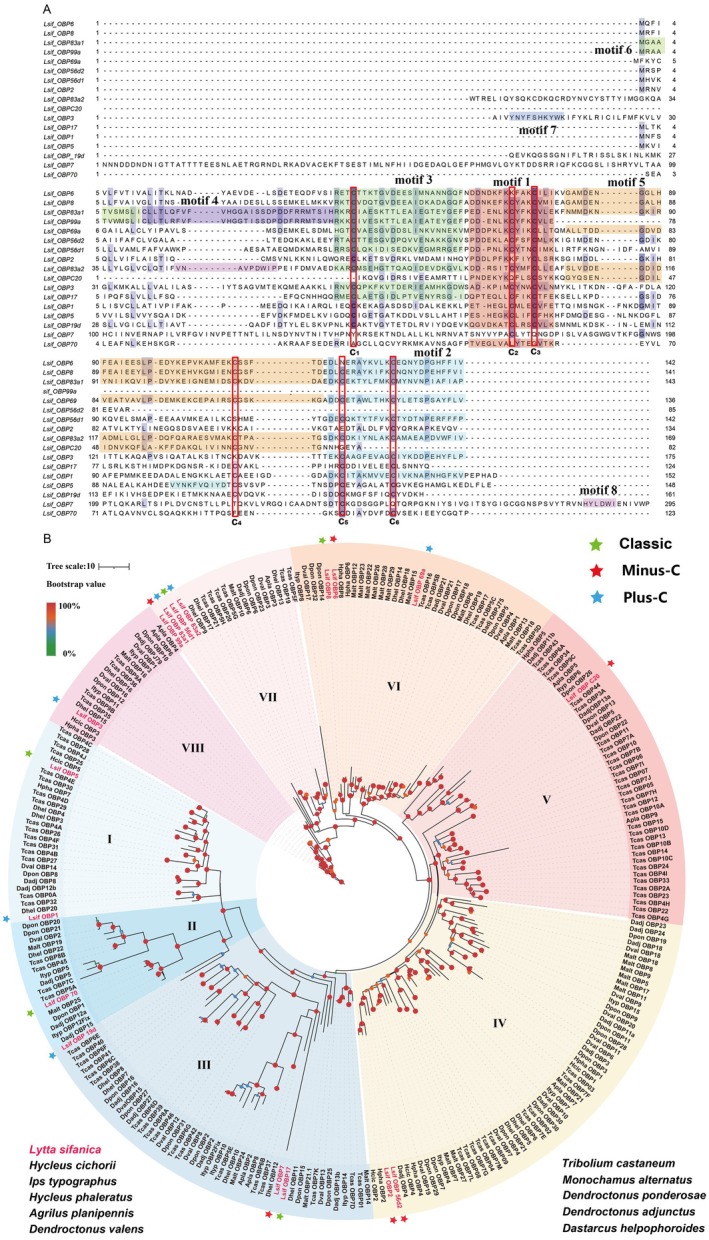
Candidate chemical analysis of OBP. (A) Multiple sequences alignment of OBPs of *L. sifanica* with other insects OBPs. Sequence alignment of 17 *L. sifanica* OBPs. Conserved cysteine residues are indicated by capital bold letters and marked with red boxes (C1–C6 and C3′ –C5′). (B) Maximum likelihood tree of the candidate OBPs predicted from the antennal transcriptome of *L. sifanica* and from several other beetles. Includes sequences from *T. castaneum, D. valens, H. cichorii, I. typographus, H. phaleratus, A. planipennis, M. alternatus, D. ponderosae, D. adjunctus, D. helpophoroides*. The distance tree was rooted by the lush orthologs. Node colors represent bootstrap support (*n* = 100) with gradient blue to red representing bs 40 to 100. Branch support was estimated using 1000 bootstrap replicates, and bootstrap values were displayed with color circles at the branch nodes in different colors.

Transcriptomic analysis of *L. sifanica* antennae identified 17 putative OBP genes, encoding proteins with an average length of 138 amino acids. The amino acid sequence identities between the predicted OBPs of *L. sifanica* and those from other beetle species in the NCBI Nr database ranged from 9.0% to 35.4%. Among the identified OBP unigenes, 11 members had full‐length ORFs and encoded a putative signal peptide at the N‐terminal region. The remaining OBP unigenes contained partial ORF sequences, for which no signal peptides were successfully predicted (Table S1: sheet 1). OBPs are typically classified into several distinct subgroups based on their cysteine residue patterns. Classic OBPs are characterized by six cysteine residues at conserved positions. In contrast, Plus‐C OBPs contain 4–6 additional cysteines compared to classic OBPs, whereas Minus‐C OBPs lack specific cysteine residues (typically the 2nd and 5th cysteines, designated C2 and C5) relative to the six conserved cysteines of classic OBPs. This classification system reflects important structural variations that significantly influence the protein folding and ligand‐binding capabilities of different OBP subfamilies (Li et al. 2015). Based on these criteria, five OBPs (*Lsif_OBP8*, *Lsif_OBP17*, *Lsif_OBP56d1*, *Lsif_OBP5*, and *Lsif_OBP70*) were identified as classic OBPs. Seven OBPs (*Lsif_OBP6*, *Lsif_OBP99a*, *Lsif_OBP56d2*, *Lsif_OBP2*, *Lsif_OBPC20*, *Lsif_OBP7*, and *Lsif_OBPC20*) and five OBPs (*Lsif_OBP83a1*, *Lsif_OBP69a*, *Lsif_OBP83a2*, *Lsif_OBP1*, and *Lsif_OBP19d*) were categorized as the Minus‐C and Plus‐C OBPs respectively (Figure 2A). All identified OBPs were further analyzed for motifs and found eight members in their family (Figure S3). Motif 1 was detected in all OBP proteins except *Lsif_OBP7*, while motif 2 and motif 3 were found in eight and ten OBPs, respectively. The remaining motifs were found in fewer than six sequences. Notably, motifs 1, 2, 3 and 5 contained highly conserved cysteine residues (C), whereas other motifs also exhibited conserved residues such as Serine (S), Tyrosine (Y), and Aspartic acid (D), indicating a relatively conserved structural domain in OBPs. Overall, the motif analysis revealed broadly conserved patterns with some variability across the OBP family in *L. sifanica* (Figure S3).

To elucidate the evolutionary relationships of OBPs in *L*. *sifanica*, the phylogenetic tree was constructed using 278 protein sequences of OBPs from 11 beetle species (Table S3). The results showed that all OBPs built a tree with eight distinct branches (I–VIII). All 17 OBPs of *L. sifanica* were distributed along various branches, and each *L. sifanica* OBP clustered with at least one ortholog from other beetle species, consistent with their functional classifications (Figure 2B). Notably, seven OBPs (*Lsif_OBP5*, *Lsif_OBP1*, *Lsif_OBP70*, *Lsif_OBP19d*, *Lsif_OBPC20*, *Lsif_OBP69a*, and *Lsif_OBP5*) clustered with their orthologs from other beetles, respectively. Additionally, *Lsif_OBP7* and *Lsif_OBP17*, *Lsif_OBP2* and *Lsif_OBP56d2*, *and Lsif_OBP6, and Lsif_OBP8* each formed separate clusters with corresponding orthologs. Interestingly, *Lsif_OBP99a*, *Lsif_OBP83a1*, *Lsif_OBP56d1*, *Lsif_OBP83a*, and *Lsif_OBP99a* were located at the root of the tree and not clustered with other homologs of beetles. These sequences may represent either evolutionarily novel genes or potential assembly artifacts resulting from sequence fragmentation.

### 3.4 Candidate Chemoseneory Proteins (CSP) Analysis

**FIGURE 3:**
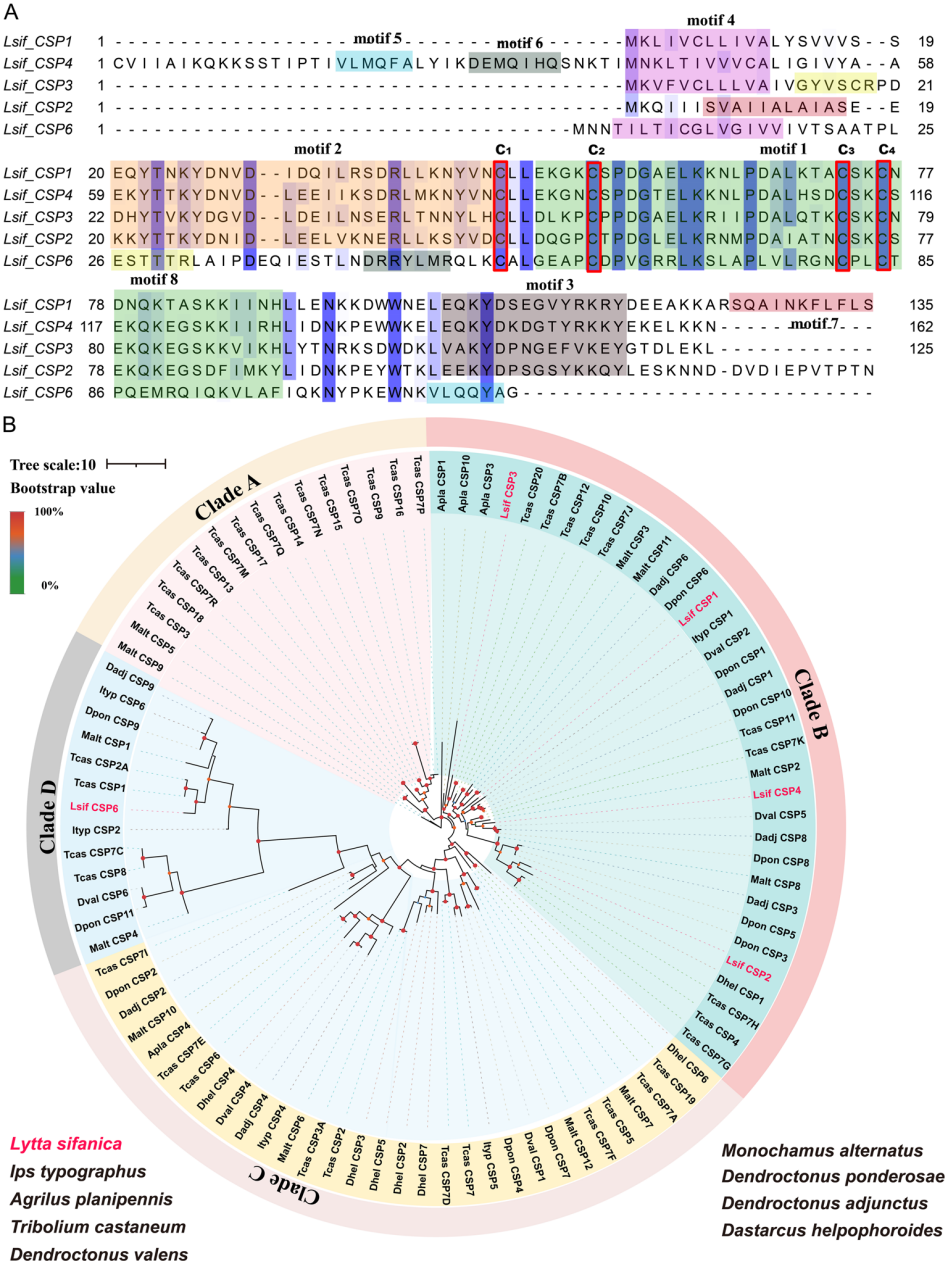
Candidate chemical analysis of CSP. (A) Multiple sequences alignment of CSPs of *L. sifanica* with other insects CSPs. Highly conserved cysteine residues are indicated by capital bold letters and marked with red boxes. (B) Maximum likelihood tree of chemosensory proteins (CSPs). Insect CSPs: *I. typographus*, *A. planipennis* , *T. castaneum* , *D. valens* , *M. alternatus* , *D. ponderosae* , *D. adjunctus* , and *D. helponphorides* were also used for the conteuction of the tree, the CSPs of *L. sifanica* were red. Branch support was estimated using 1000 bootstrap replicates, and bootstrap values were displayed with color circles at the branch nodes. The scale bar indicates the expected number of amino acid substitutions per site.

A total of five CSPs (*Lsif_CSP1*, *Lsif_CSP2*, *Lsif_CSP3*, *Lsif_CSP4*, and *Lsif_CSP6*) were identified in *L. sifanica* antennal tissue, ranging from 117 to 163 amino acids. Four members (*Lsif_CSP1*, *Lsif_CSP2*, *Lsif_CSP3*, and *Lsif_CSP6*) contained complete open reading frames (ORFs) and N‐terminal signal peptides, while *Lsif_CSP4* was partially assembled due to a missing 5′ terminus (Table S1: sheet 2). All identified CSPs exhibited the characteristic highly conserved pattern of four cysteine residues (Cys‐X6‐Cys‐X18‐Cys‐X 2‐Cys, where X represents any amino acid), which is characteristic of the CSP gene family (Figure 3A). All candidate CSP genes had four conserved cysteines in the corresponding positions and a conserved OSD domain (InterPro: IPR005055). Motif analysis identified eight conserved motifs in *L. sifanica* CSPs. Motif 1, containing three cysteine residues, was present in all family members, consistent with the conserved structure of insect CSPs. Motif 2 appeared in four of five CSPs, with both motifs showing high sequence conservation. The remaining motifs displayed partial conservation among family members (Figure S4). Motif analysis results clearly provide us with the structural characteristics of OBP sequences, and different conserved segments.

A maximum‐likelihood (ML) phylogenetic tree was reconstructed using 103 CSP amino acid sequences from nine coleopteran species (*L. sifanica*, *I. typographus*, *A. planipennis* , *T. castaneum* , *D. valens* , *M. alternatus* , *D. ponderosae* , *D. adjunctus* , and *D. helpophoroides*) (Table S3). All CSPs were clustered into four distinct clades (designated A–D), with five *L. sifanica* CSPs distributed in Clades B and D, each clustering with at least one ortholog from other beetle species. Among these five candidate CSPs, two members (*Lsif_CSP6* and *Lsif_CSP3*) clustered together with *T. castaneum* CSPs (*Tcas_CSP1* and *Tcas_CSP20*) separately. The remaining two members (*Lsif_CSP1* and *Lsif_CSP2*) of *L. sifanica* were clustered together with *D. ponderosae* (*Dpon_CSP1* and *Dpon_CSP20);* only *Lsif_CSP4* was clustered together with *Dval_CSP5* (Figure 3B). Thus, the CSPs of *L. sifanica* we found are homologous to CSPs in beetles. Interestingly, there are no CSP members of *L. sifanica* located on branches A and C, which may suggest that some members are lacking in evolutionary branches, in which other beetles' CSPs are expanded. This phylogenetic distribution pattern may reflect functional diversification of CSP genes during coleopteran evolution.

### 3.5 Candidate Gustatory Receptors (GRs) Analysis

Thirteen candidate GRs were identified in the antennal transcriptome of *L. sifanica*, with an average sequence length of 175 amino acids. Only five GRs contained a complete open reading frame (ORFs), while the remaining sequences were incomplete due to a lack of a 5′ or 3′ terminus. Notably, we observed significant length variation among the encoded proteins, with some GRs exhibiting substantially longer ORFs than other family members (Table S1: sheet3). Motif analysis revealed three conserved domains in *L. sifanica* GRs. while motif 1 was present in all GRs except *Lsif_GR7*. Motif 2 and 3 were each detected in only two sequences. This restricted conservation pattern suggests relatively low sequence similarity among *L. sifanica* GR family members, potentially indicating functional diversification within this receptor family (Figure S5).

**FIGURE 4:**
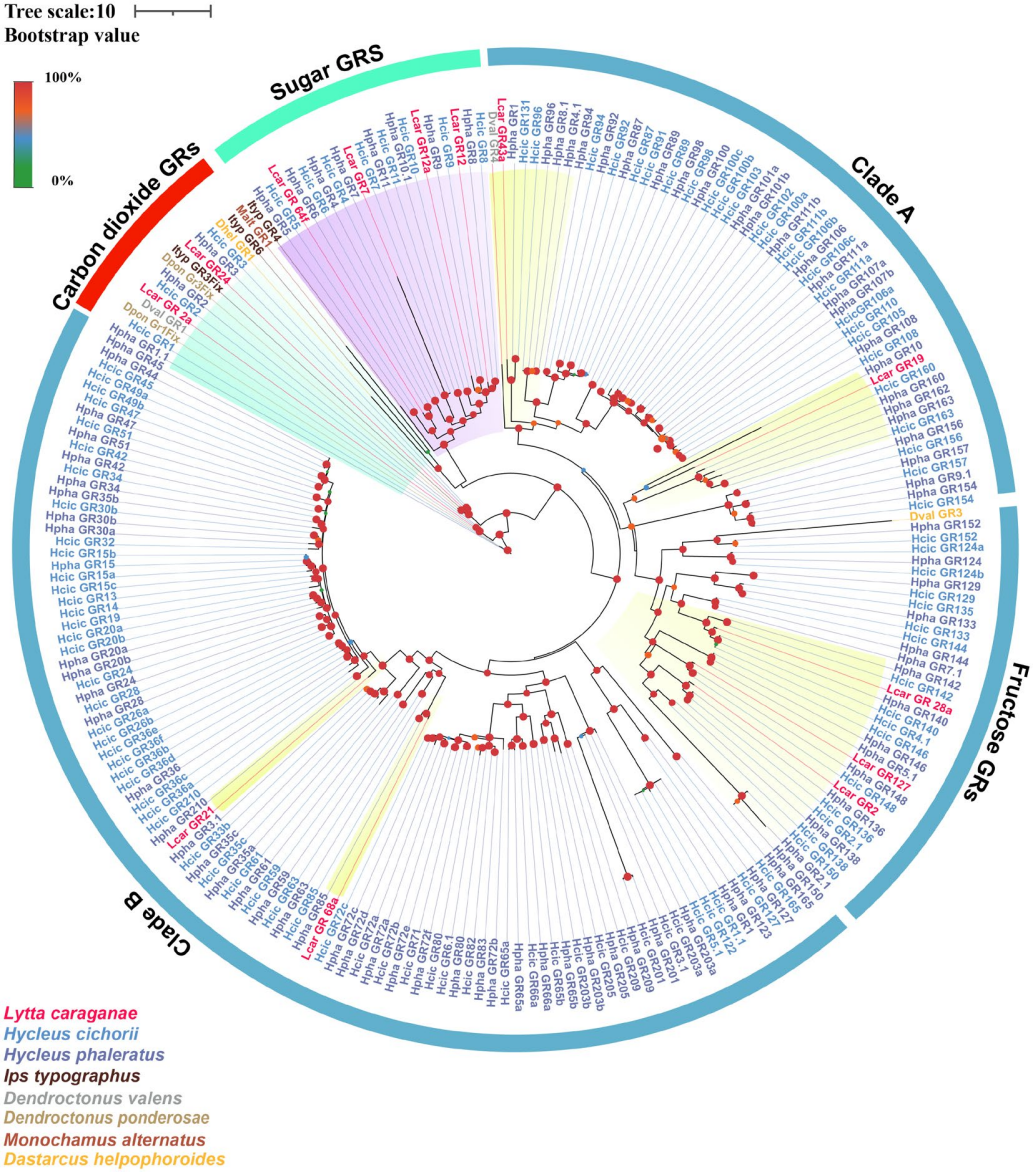
Candidate chemical analysis of GR. Maximum likelihood Phylogeny based on the protein sequence of the candidate GRs. Red: *L. sifanica*, Blue: *H. cichorii*, Purple: *H. phaleratus*, Brown: *I. typographus*. The stability of the nodes was assessed by bootstrap analysis with 1000 replications. The scale bar indicates the expected number of amino acid substitutions per site.

A GR phylogenetic tree based on 224 protein sequences from eight beetles (*L. sifanica, H. cichorii, H. phaleratus, M. alternatus, D. ponderosae, D. valens, I. typographus*, and *D. helpophoroides*) was then constructed. All the GRs of *L. sifanica* were clustered with their orthologs from other beetles into five distinct clades (Table S3). Notably, four GRs (*Lsif_GR12*, *Lsif_GR12a, Lsif_GR64f*, and *Lsif_GR7*) grouped within the conserved sugar receptor clade. Three members (*Lsif_GR2, Lsif_GR127*, and *Lsif_GR28a*) formed a distinct cluster with known fructose receptors. while *Lsif_GR24* and *Lsif_GR2a* clustered with other known carbon dioxide GRs and formed a clade. The remaining three GRs showed phylogenetic relationships with expanded GR subfamilies in other beetle species (Figure 4). This phylogenetic distribution demonstrates that *L. sifanica* possesses GRs representing multiple functional classes, though with fewer lineage‐specific expansions compared to other Meloidae species. The conserved clustering patterns with characterized beetle GRs suggest maintained functional specialization during evolution.

### 3.6 Candidate Ionotropic Receptors (IR) Analysis

A total of thirteen putative ionotropic receptors (IRs) were identified from the antennal transcriptome of *L. sifanica*, with an average predicted protein length of 256 amino acids. Among them, only two sequences contained a complete open reading frame (ORF), while the remaining IRs were incomplete due to missing 5′ or 3′ terminus. Except for *Lsif_IR2a*, no signal peptides were predicted in the other IR sequences (Table S1: sheet 5). The transmembrane domain prediction indicated that only *Lsif_IR_56e* was predicted to possess four transmembrane domains. Motif analysis revealed six conserved motifs within the IR family of *L. sifanica*. Nevertheless, these motifs were only partially present in most IR members and exhibited variable arrangements. Except for *Lsif_IR19*, which contained all six motifs, the other IRs possessed fewer than four of the six motifs, suggesting low evolutionary conservation within the IR family of *L. sifanica* (Figure S6).

**FIGURE 5:**
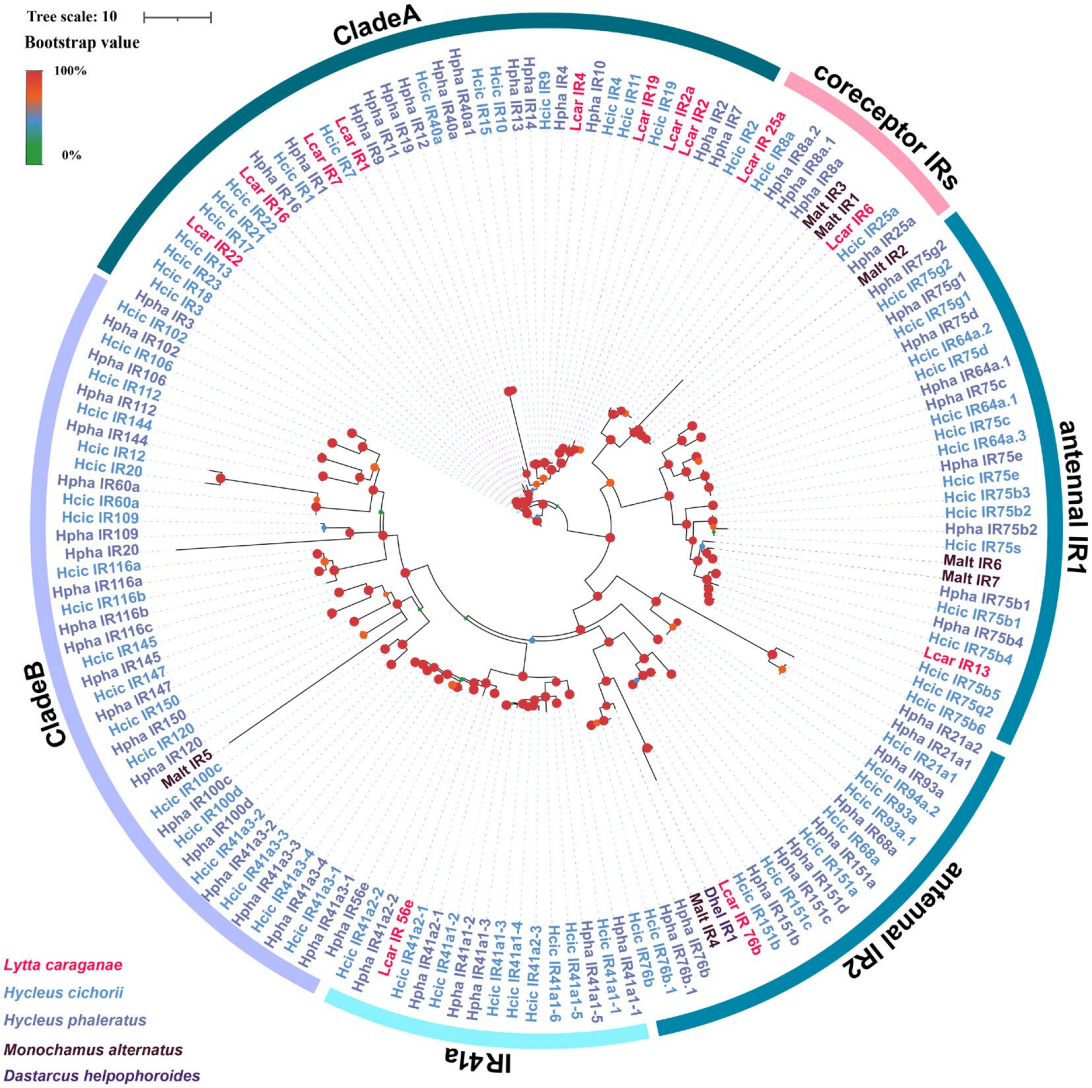
Candidate chemical analysis of IR. Maximum likelihood tree for putative IRs from *Lytta sifanica* and other beetles. Lsif: *L. sifanica*, Hcic: *H. cichorif*, Hpha: *H. phaleratus*, Malt: *M. alternatus*, Dhel: *D. helpophoroides*. Branch support was estimated using 1000 bootstrap replicates, and bootstrap values were displayed with color circles at the branch nodes. The scale bar indicates the expected number of amino acid substitutions per site.

The phylogenetic tree was constructed using IR sequences from *L. sifanica* and four other beetle species (*H. Cichorii, H. phaleratus, D. helophoroides*, and *M. alternatus* ), and revealed six major clades (Table S3). Two IRs (*Lsif_IR_25a* and *Lsif_IR6*) of *L. sifanica* clustered separately with orthologs of coreceptor IRs from other beetles. *Lsif_IR13* and *Lsif_IR76b* grouped with their respective orthologs from other beetles within two distinct antennal IR clades. *Lsif_IR56e* clustered with orthologs of other beetles in the clades 41a expression IRs. The other nine IR members of *L. sifanica* showed strong phylogenetic affinity with coleopteran orthologs, forming a distinct clade (Clade A) basal to the phylogenetic tree (Figure 5). Maximum likelihood analysis confirmed these genes as authentic IR family members, revealing a notable expansion shared among beetles.

### 3.7 Candidate Odorant Receptors (ORs) Analysis

A total of seventeen putative ORs were analyzed from the antennal transcriptome of *L. sifanica*. Nine ORs possessed complete open reading frames (ORFs), encoding proteins ranging from 107 to 478 amino acids in length, while the remaining sequences were incomplete due to missing 5′ or 3′ terminus. One OR co‐receptor (*Lsif_ORco*) was identified as full‐length ORFs that encode 228–478 amino acids (Table S1: sheet5). The predicted transmembrane structure revealed that six ORs were predicted to contain six to seven transmembrane domains (TMDs), a characteristic feature of typical insect ORs (Cheema et al. 2021). Nevertheless, the remaining sequences showed less than five TMDs, which may be caused by incomplete ORFs (Figure S9). Motif analysis identified eight conserved domains in the ORs family of *L. sifanica*. Motifs 1, 2, and 3 were the most conserved and were present in all nine complete ORs. Interestingly, five OR members (*Lsif_OR*1, *Lsif_OR4*, *Lsif_OR10*, *Lsif_OR14*, and *Lsif_OR17*) showed 1–2 motifs in their sequences, possibly due to the lack of a 5′ or 3′ terminus (Figure S7). The motif locations suggested that the C terminus of the OR family in *L. sifanica* is more conservative.

**FIGURE 6:**
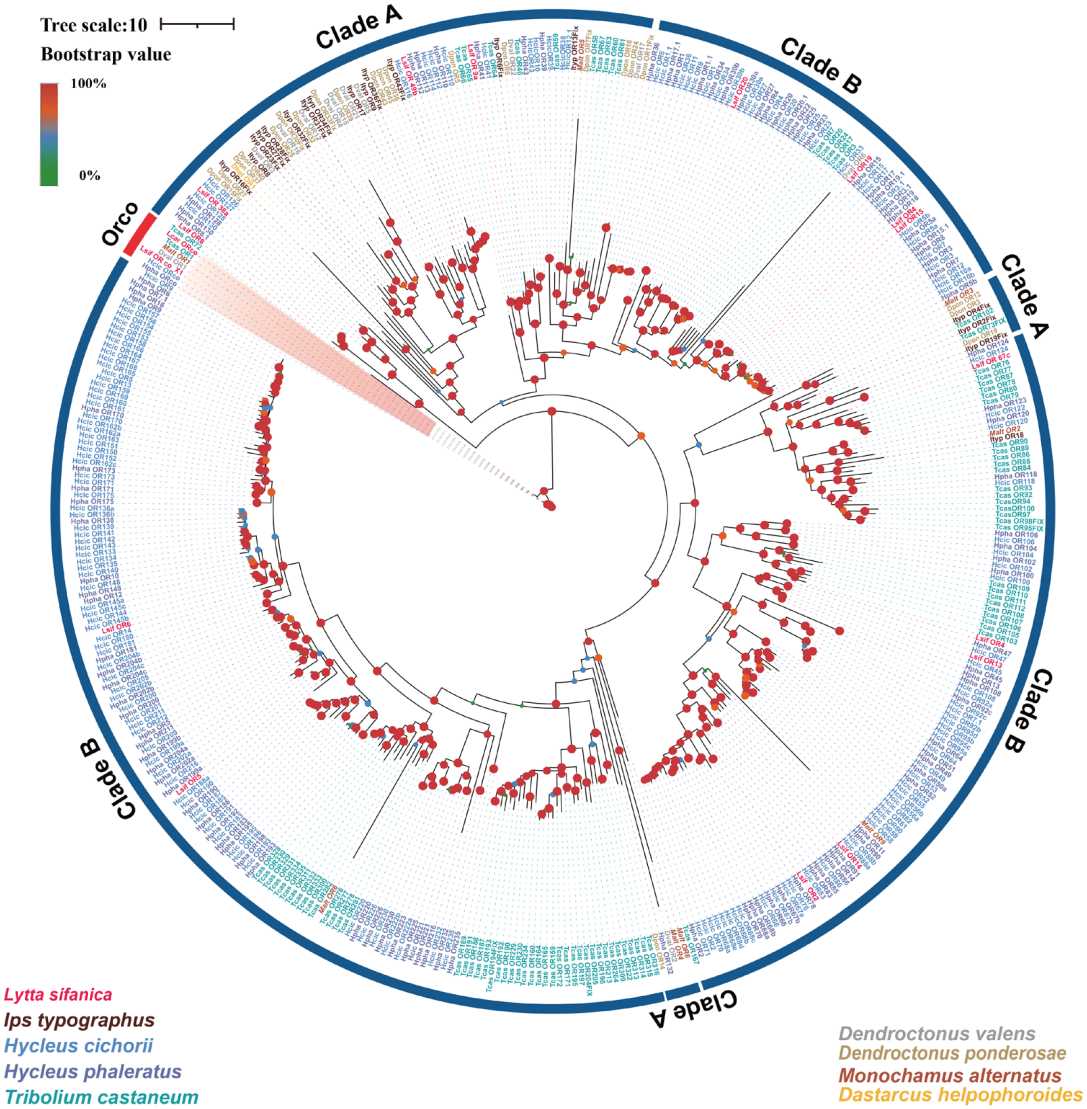
Candidate chemical analysis of OR. Maximum likelihood tree of putative ORs from *L. sifanica* and other beetles. Branch support was estimated using 1000 bootstrap replicates, and bootstrap values were displayed with colored circles at the branch nodes. The scale bar indicates the expected number of amino acid substitutions per site.

A maximum‐likelihood phylogenetic tree was constructed using the identified ORs in *L. sifanica* together with known OR sequences from eight other beetle species, including *I. typographus*, *H. cichorii*, *H. phaleratus* , *T. castaneum* , *D. valens* , *D. ponderosae* , *D. helophoroides*, and *M. alternatus* (Table S3). The analysis revealed that 444 proteins were grouped into six major evolutionary clades (I–VI). Each OR of *L. sifanica* clustered with its orthologs from other beetles, forming distinct branches within the tree. Notably, the two ORco (*Lsif_ORco* and *Lsif_ORco_X1*) of *L. sifanica* formed a distinct clade VI at the phylogenetic tree root with orthologs from other beetles. This basal clade exhibited high sequence conservation and early evolutionary divergence from other OR members, a pattern consistent with observations in other Coleoptera species (Gu et al. 2015). Several lineage‐specific clades were also identified, comprising expanded OR lineages within individual beetle species such as *T. castaneum* and *H. cichorii* (Figure 6). In summary, the ORs identified in *L. sifanica* were homologous to those of other beetles, but no expansion of OR family members was detected in this species.

### 3.8 Candidate Sensory Neuron Membrane Proteins (SNMP) Analysis

Five candidate SNMPs were identified from the antennal transcriptome of *L. sifanica*. Four members (*Lsif_SNMP1*, *Lsif_SNMP2*, *Lsif_SNMP3*, and *Lsif_SNMP4*) possessed complete open reading frames (ORFs) encoding 154 to 568 amino acids (Table S1: sheet 6). Motif analysis revealed that five SNMP proteins of *L. sifanica* possessed eight conserved motifs. Each motif was unevenly distributed among different SNMP family members. *Lsif_SNMP1*, *Lsif_SNMP2*, and *Lsif_SNMP4* contained all eight motifs, whereas *Lsif_SNMP3* and *Lsif_SNMP5 contained* fewer than three motifs, likely due to incomplete ORFs (Figure S8).

**FIGURE 7:**
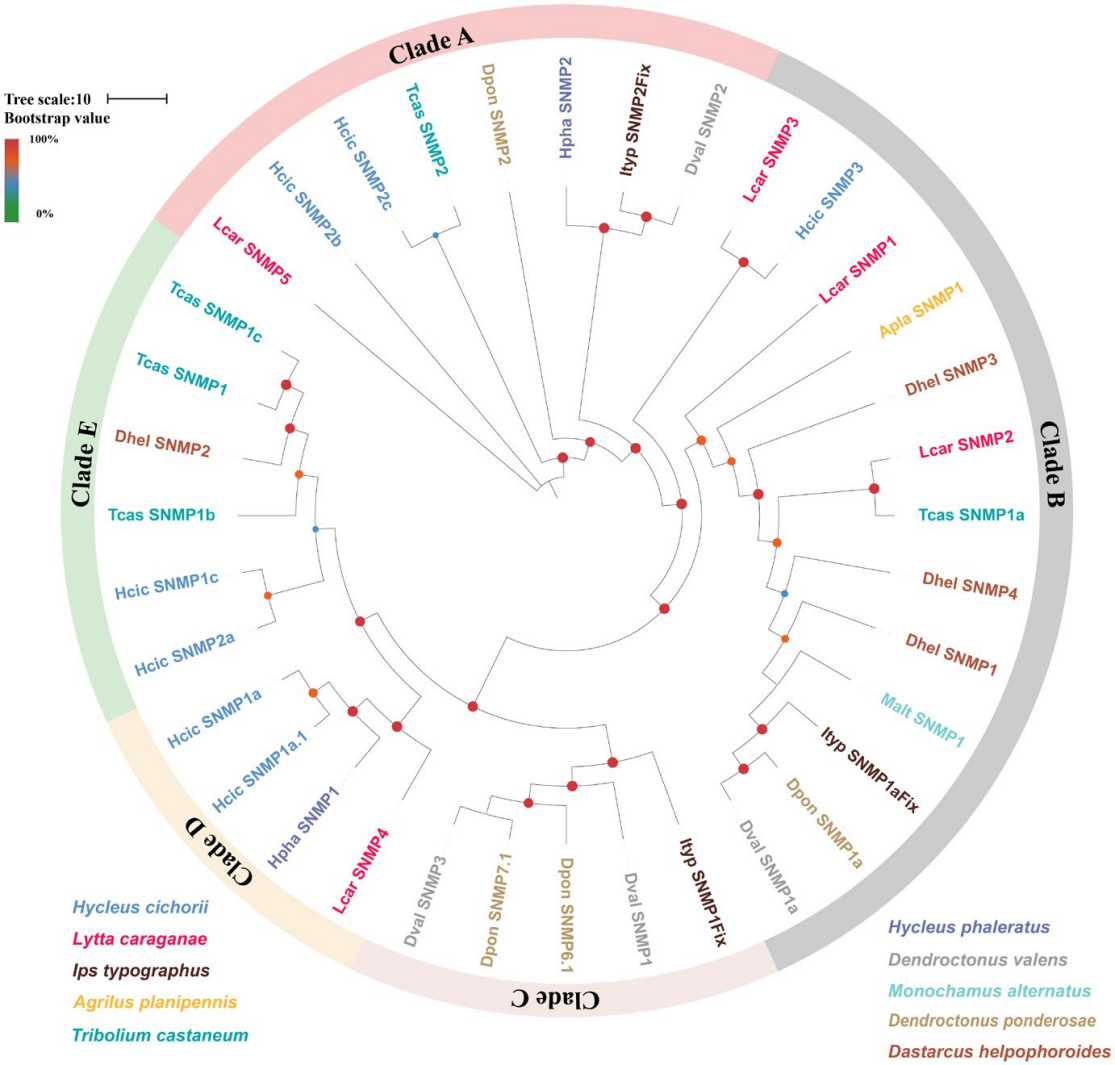
Candidate chemical analysis of SNMP. Maximum likelihood tree of the SNMPs from the antennal transcriptome of *L. sifanica* and from several other beetles. Include sequences from *H. cichorii, I. typographus, A. planipennis, T. castaneum, H. phaleratus, D. valens, M. alternatus, D. ponderosae, D. helpohoroides*. Branch support was estimated using 1000 bootstrap replicates, and bootstrap values were displayed with color circles at the branch nodes. The scale bar indicates the expected number of amino acid substitutions per site.

To investigate the evolutionary relationships of all putative SNMPs in *L. sifanica* with those of other beetles, a phylogenetic tree was constructed using 36 protein sequences from four representative beetle species (*H. cichorii*, *T. castaneum* , *A. planipennis*, and *I. typographus*) (Table S3). Five distinct clades (Clade A–E) were clustered in the phylogenetic tree. The five SNMPs *L. sifanica* SNMPs clustered with orthologs from other beetle species and were distributed across three clades (Clade A, B, D). Among them, *Lsif_SNMP3, Lsif_SNMP1*, and *Lsif_SNMP4* were orthologs of *Hcic_SNMP3, Apla_SNMP1*, and *Tcas_SNMP1a*, respectively (Figure 7). In summary, the SNMP gene family of *L. sifanica* exhibits high evolutionary conservation and comprises a slightly larger number of members compared with those of other blister beetles.

### 3.9 Tissue‐Specific Expression Patterns of Olfactory‐Related Protein in *L. Sifanica*

**FIGURE 8:**
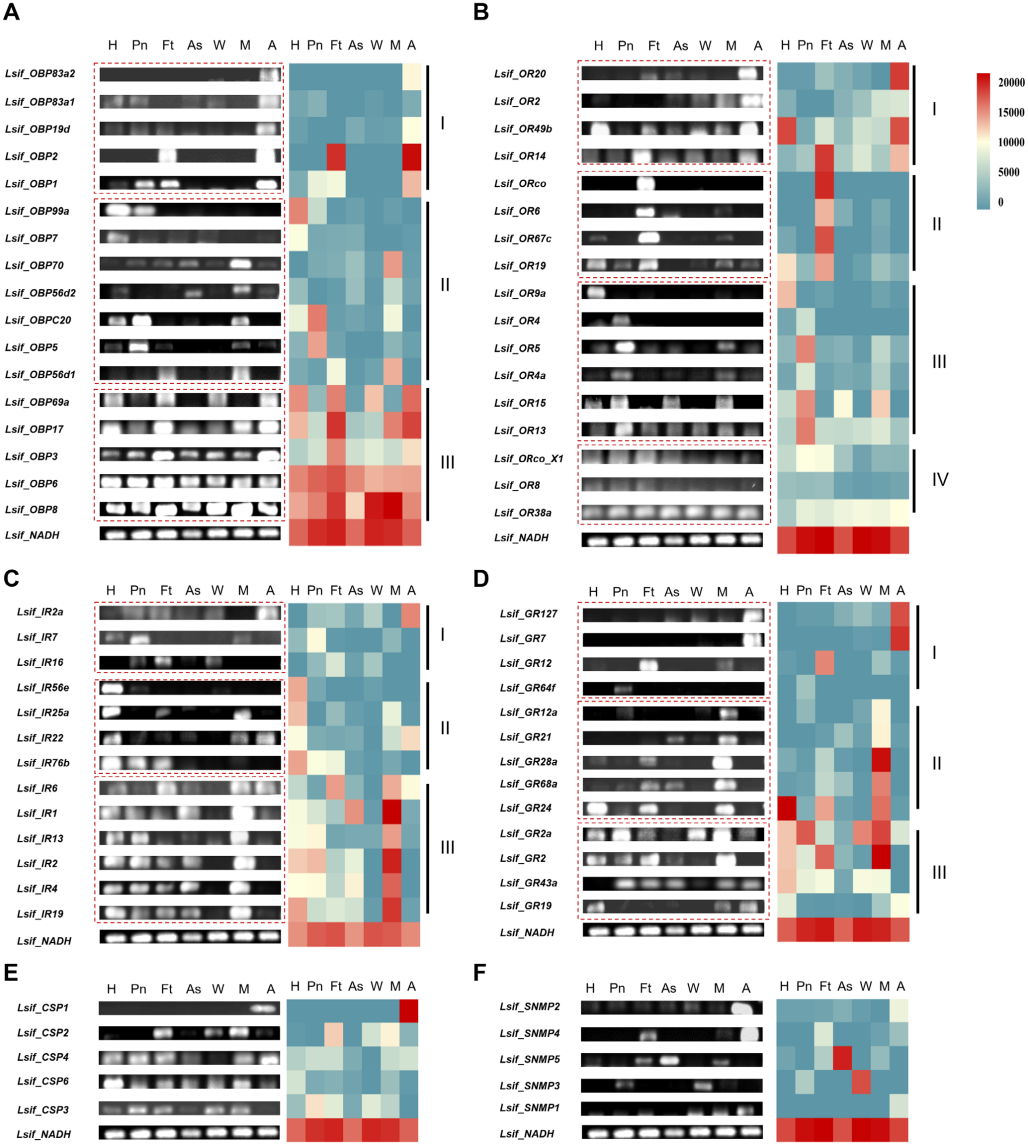
Tissue‐Specific Expression of olfactory related proteins. Tissue‐specific expression of *Lsif_OBPs*, *Lsif_SNMPs, Lsif_CSPs, Lsif_ORs, Lsif_GRs, Lsif_ORs* as measured by RT‐PCR. (A) OBPs, (B) ORs, (C) IRs, (D) GRs, (E) CSPs, (F) NMPs. Tissue‐specific expression patterns of seventy‐seven candidate genes analyzed by RT‐PCR, Hot‐map indicates expression patterns of genes in seven locust tissues including Head (H), Pronotum (Pn), Foreleg tarsus (Ft), Abdomen Skin (As), wings (W), Mouthparts (M), antennae (A). Red indicates high expression patterns, whereas blue indicates low expression levels.

To further illustrate the expression profiles of the identified olfactory‐related proteins, the relative expression levels (FPKM values) from the antennal transcriptome were first analyzed for all identified members of olfactory‐related gene families. Subsequently, RT‐PCR was employed to examine the expression patterns of these genes across multiple tissues of *L. sifanica*, including the antennae, mouthparts, head (without antennae and mouthparts), pronotum, foreleg tarsus, abdomen skin, and wings. For OBP proteins, RT‐PCR confirmed that five OBPs (*Lsif_OBP83a2*, *Lsif_OBP2*, *Lsif_OBP19d*, *Lsif_OBP83a1*, and *Lsif_OBP1*) were predominantly expressed in the antennae. Among them, *Lsif_OBP83a2*, *Lsif_OBP83a*1, and *Lsif_OBP19d* showed high expression levels, with FPKM values ≥ 1000 (Figure S10e). *Lsif_OBP2* and *Lsif_OBP1* demonstrated high expression in the foreleg tarsus and pronotum, respectively. Four members (*Lsif_OBP99a*, *Lsif_OBP7*, *Lsif_OBPC20*, and *Lsif_OBP5*) were mainly expressed in the head or pronotum, while three members (*Lsif_OBPC70*, *Lsif_OBP56d2*, and *Lsif_OBP56d1*) were specifically expressed in the mouthparts. *Lsif_OBP69a* and *Lsif_OBP17* were specifically and exclusively expressed in four tissues (head, foreleg tarsus, antennae, and mouthparts or wings). The remaining OBPs, including *Lsif_OBP3* were abundantly expressed across all analyzed tissues (Figure 8A). Overall, tissue‐specific expression analysis revealed that the OBP family members of *L. sifanica* can be expressed in multiple tissues, but antennae followed by the mouthparts are the dominantly expressed tissues, in which some members are specifically expressed.

For OR family, RT‐PCR proved that four members (*Lsif_OR20*, *Lsif_OR2*, *Lsif_OR49b*, and *Lsif_OR14*) were almost exclusively expressed in antennae. Although most of these genes showed relatively low transcript levels (FPKM < 2), *Lsif_OR14* exhibited substantially higher expression (FPKM = 42.15) (Figure S10f). Tissue‐specific expression analysis revealed distinct patterns among *L. sifanica* ORs (Figure 8B). *Lsif_OR49b* and *Lsif_OR14* also exhibited higher expression in the head and foreleg tarsus, respectively. Notably, four members (*Lsif_OR6*, *Lsif_OR67c*, *Lsif_OR19*, and *Lsif_ORco*) were almost exclusively expressed in the foreleg tarsus and *Lsif_OR19* also displayed notable expression in the head. Among them, *Lsif_OR19* and *Lsif_ORco* also showed relatively high FPKM values in antennae with 37.40 and 281.57, respectively. Interestingly, the canonical *Lsif_ORco* transcript exhibited predominant expression in the foreleg tarsus, while its splice variant *Lsif_ORco_X1* showed a relatively broad expression pattern across multiple tissues. *Lsif_OR9a* was almost exclusively expressed in the head, whereas five OR genes *(Lsif_OR4, Lsif_OR4a, Lsif_OR15, Lsif_OR13, and Lsif_OR5)* exhibited elevated expression in the pronotum. The remaining OR members were constitutively expressed across multiple tissues (Figure 8B). Overall, these results demonstrate that the ORs in *L. sifanica* were expressed in multiple tissues, with the antennae and foreleg tarsus being the primary sites of specific expression.

Expression profiling of the IR family revealed distinct tissue‐specific patterns in *L. sifanica*. Three IRs (*Lsif_IR2a*, *Lsif_IR7*, and *Lsif_IR16*) exhibited distinct tissue‐specific expression in the antennae, pronotum, and foreleg tarsus, respectively. Three other IRs (*Lsif_IR56e*, *Lsif_IR22*, and *Lsif_IR76b*) showed higher expression in the head. In addition, *Lsif_IR*25a, *Lsif_IR*22 and *Lsif_IR76b* were also highly expressed in mouthparts and antennae or pronotum and foreleg tarsus. The remaining IR members showed higher expression in the mouthparts and also constitutive expression in multiple tissues (Figure 8C). However, only three members (*Lsif_IR6*, *Lsif_IR76b*, and *Lsif_IR25a*) were highly expressed in the antennae of *L. sifanica*, whereas the other IR members showed low expression levels (FPKM < 10) (Figure S10b). Overall, IRs of *L. sifanica* are broadly expressed across multiple tissues, with the highest expression observed in the mouthparts, and several members exhibiting head‐specific expression.

Within the GR family, *Lsif_GR127* and *Lsif_GR7* were almost exclusively expressed in the antennae, despite having low FPKM values below 2. *Lsif_GR12* and *Lsif_GR65f* were mainly expressed in the foreleg tarsus and pronotum, respectively. Five GR members (*Lsif_GR12a*, *Lsif_GR21*, *Lsif_GR28a*, *Lsif_GR68a*, and *Lsif_GR24*) were mainly expressed in the mouthparts, although *Lsif_GR24* was also highly expressed in the head and pronotum. The remaining GRs were widely expressed across multiple tissues. Almost all GR members of *L. sifanica* showed low expression levels, with FPKM values below 10 (Figure S10f). Totally, GRs in *L. sifanica* were expressed in multiple tissues, with predominant expression observed in the mouthparts (Figure 8D).

All CSP members showed high expression in the antennae of *L. sifanica* (Figure S10d). For example, *Lsif_CSP2*, *Lsif_CSP3*, and *Lsif_CSP4* showed FPKM values greater than 300. However, RT‐PCR further confirmed that *Lsif_CSP1* was almost exclusively expressed in the antennae. *Lsif_CSP2* was more highly expressed in other tissues, including mouthparts and foreleg tarsus. The other members (*Lsif_CSP4*, *Lsif_CSP6*, and *Lsif_CSP3*) showed constitutive expression in multiple tissues (Figure 8E). These results suggest that although CSPs of *L. sifanica* show high expression in the antennae, they are not exclusively expressed in this tissue.

RT‐PCR further validated that both *Lsif_SNMP2* and *Lsif_SNMP4* were almost exclusively expressed in the antennae, with high FPKM values ≥ 300 (Figure S10a). *Lsif_SNMP5* and *Lsif_SNMP3* were mainly expressed in the abdominal integument and wings, respectively. *Lsif_SNMP1* was expressed in multiple tissues (Figure 8F). Overall, these results demonstrate that SNMPs of *L. sifanica* show high expression in the antennae.

## 4 Discussion

**FIGURE 9:**
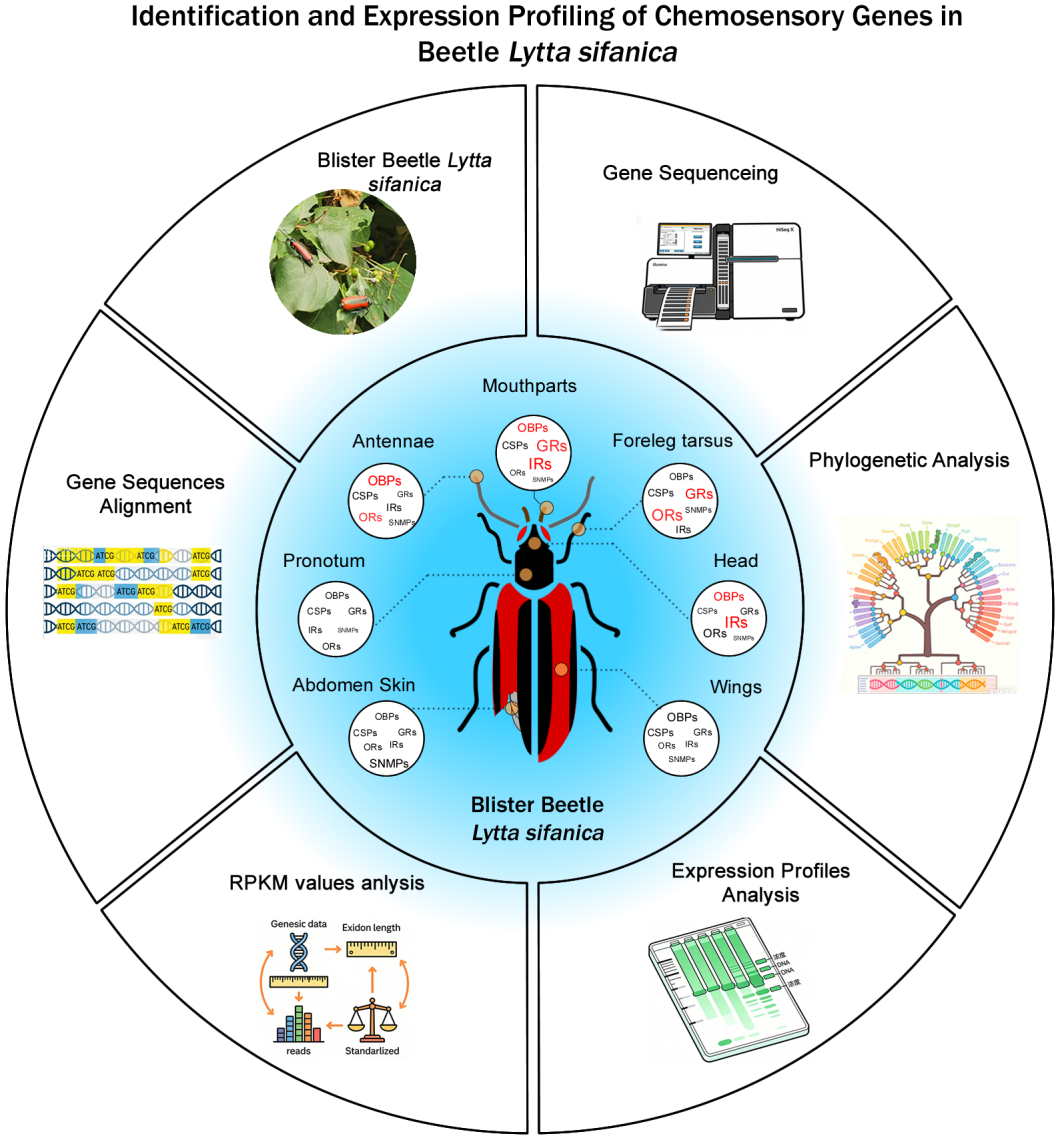
The summary of Identification and Expression Profiling of Chemosensory Genes in Beetle *Lytta sifanica*.

The olfactory—related genes play crucial roles in manipulating multiple insect behaviors (Asahina et al. 2008), in particular, the following proteins are essential in chemosensory reception: OBPs, CSPs, SNMPs, ORs, GRs, and IRs (Leal 2013). They are capable of detecting chemical signals in the environment. These signals are conveyed to the nervous system via olfactory‐related genes, where they regulate key behaviors, including orientation, mating, foraging, and predator avoidance (Zhang et al. 2021). For example, in the above study on olfactory genes in flies, olfactory information is transmitted by a chain of multiple genes. Importantly, the high expression of certain olfactory receptor genes may be closely associated with the efficient odor recognition ability of *L. sifanica*. In *Curculio Dieckmanni*, *Cdie_OR13* and *Cdie_OR15* exhibit pronounced female‐biased expression (Ma et al. 2022). Therefore, these olfactory receptor genes are essential for locating mates and oviposition sites. Understanding the genetic basis of these olfactory genes is crucial for elucidating the behavior of these insects. In this study, we investigated the genetic basis of the chemosensory system in *L. sifanica*. Based on the antennal transcriptome, 70 related proteins were identified, including members of the OBP, CSP, SNMP, OR, IR, and GR families. Phylogenetic analysis revealed evolutionary relationships between these genes and their orthologs in other blister beetles and Coleoptera. Surprisingly, not all olfactory genes were highly expressed in the antennae. Instead, many olfactory gene members showed tissue‐specific high expression in multiple tissues, including the mouthparts, head, and foreleg tarsus. These genetic features and unique expression patterns may reflect the behavioral and ecological traits of *L. sifanica*. Moreover, the phylogenetic trees constructed in this study demonstrated that the olfactory—related proteins of *L. sifanica* had similar cluster expression as those in other beetles. Thus, we could speculate upon the functional characteristics of *L. sifanica* based on comparisons with those of other known species as follows. The summary of this study for this beetle were showed in Figure 9.

OBPs play crucial roles in insect olfaction by facilitating the transport of hydrophobic odorants through the sensillar lymph and mediating interactions between environmental chemical cues and olfactory receptors(Lechuga‐Paredes et al. 2023). Their functions typically rely on three stable disulfide bonds formed by six highly conserved Cys residues, which are characteristic features used to identify members of this family. A total of seventeen OBPs were identified in the antennal transcriptome of *L. sifanica* through homology‐based searches and were further classified into subclades consisting of six Minus‐C, six Plus‐C and five Classic‐C members. Motif analysis revealed characteristic OBP features and identified highly conserved residues present in all OBPs. These results are generally consistent with the characteristics observed in other Coleopteran species. However, compared to other beetles, the antennal transcriptome of *L. sifanica* lacks dimer‐OBP genes, suggesting that this OBP subtype is likely expressed in other tissues rather than in the antennae of blister beetles. Comparative genomic analysis with *I. typographus* and *D. ponderosae* (Andersson et al. 2013) revealed that the *L. sifanica* beetle possesses an additional Classic‐C subtype, suggesting potential functional specialization in this species. Previous studies have established that Minus‐C OBPs are more abundant in insects, whereas Plus‐C OBPs are more prevalent in more evolutionarily derived species (Vieira and Rozas 2011; Spinelli et al. 2012). The abundance of these subtypes in the blister beetle transcriptome is generally consistent with previous findings, and studies of blister beetles and other insects indicate that Minus‐C OBPs are the most prevalent (Capinera et al. 1985). The antennal‐specific expression pattern of these OBP subtypes indicates their potential functional specialization in olfactory signal transduction. Phylogenetic analysis of OBPs from several beetles showed that the Minus‐C OBP in this blister beetle forms an independent branch with homologs from other beetles. In contrast, Classic‐C and Plus‐C OBPs exhibit lower sequence conservation and are positioned closer to the base of the phylogenetic tree, reflecting the evolutionary conservation of Minus‐C OBP subtypes. Expression profiling revealed that OBP subtype classification does not strictly correlate with tissue specificity in *L. sifanica*. Notably, OBPs from each subtype in *L. sifanica* are predominantly expressed in the antennae, suggesting that the antennae are a key tissue for OBP expression. For instance, in *Tenebrio molitor* , approximately half of the OBPs are predominantly expressed in the antennae (Liu et al. 2015). Additionally, many OBPs are specifically expressed in the labial palps, whereas others are expressed broadly across multiple tissues. Recent studies have also demonstrated that OBPs function in non‐chemoreceptive tissues, such as pheromone glands, where they participate in pheromone release (Jacquin‐Joly et al. 2001; Dani et al. 2011). OBPs expressed in non‐antennal tissues merit further functional investigation.

CSPs are a conserved family of small binding proteins that can bind pheromone compounds (Liu et al. 2020). Five CSP members were identified in the antennae of *L. sifanica*. The high sequence similarity observed in BLASTp analyses demonstrates that CSPs are highly conserved proteins in insects. Compared with other Coleopteran species, *L. sifanica* has fewer CSP genes (five) than *Leptinotarsa decemlineata* (15 CSPs) and *Glnea cantor* (14 CSPs) (Liu et al. 2012), but a similar number to *Callosobruchus maculatus* (Wang et al. 2017). This indicates that, compared to other coleopteran beetles, the CSP (chemosensory protein) family in *Lytta vesicatoria* has undergone significant gene contraction. This is by no means a simple “defect” but rather likely represents a key adaptive evolutionary event, suggesting that the chemosensory proteins in *Lytta vesicatoria* have become highly specialized and streamlined—a hallmark of adaptive evolution. The amino acid sequences of *L. sifanica* CSPs revealed a typical four‐cysteine motif at conserved positions, conforming to the CSP model of C1‐X6–8‐C2‐X16–21‐C3‐X2‐C4 (X represents any amino acid) (Dippel et al. 2014). Motif analysis revealed that the second, third, and fourth cysteines are located in Motif 1, whereas the first cysteine is located in Motif 2. This is consistent with the multiple sequence alignment of *L. sifanica* CSPs, indicates that these genes are highly conserved in structure and suggesting their critical roles in biological functions reported in other beetles (Wanner et al. 2004). Phylogenetic analysis revealed distinct evolutionary patterns among *L. sifanica* CSPs, with five members distributed across divergent clades. Notably, *Lsif_CSP1*, *Lsif_CSP*2, *Lsif_CSP*3, and *Lsif_CSP*4 cluster near the root of the phylogenetic tree, indicating that these CSP genes are evolutionarily conserved (Capinera et al. 1985). These genes typically have relatively general functions, retaining most of their original roles. They are likely widely present across organisms and have undergone minimal divergence or adaptation (Wang et al. 2017). In contrast, *Lsif_CSP6* clusters near the terminal branches of the phylogenetic tree (Figure 3B), suggesting that it is a more specialized gene derived from ancestral forms. This gene may have undergone more mutations or adaptive changes, enabling it to perform specific functions. Such specialization may be accompanied by structural or functional modifications (Wanner et al. 2004). These observations are further supported by tissue‐specific expression analyses. *Lsif_CSP3* and *Lsif_CSP4* are expressed in multiple tissues, indicating their broad functional roles. In contrast, *Lsif_CSP6* is highly expressed in the head and mouthparts, suggesting that it may play roles in prey capture and host plant detection. Nevertheless, *Lsif_CSP1* exhibits antenna‐specific expression, indicating a key role in pheromone detection via the antennae.

GRs have been identified in various insect species. They are mostly expressed in gustatory receptor neurons in taste organs and are involved in the detection of sugars, bitter compounds, CO_2_ and some pheromones (Ma et al. 2019). Thus, insects often evolved diverse GR family members to fulfill these functions. In this study, a total of thirteen GR family members were identified in the antennae of *L. sifanica*. These GR sequences exhibit a certain degree of sequence conservation within the species, as evidenced by motif analysis. Specifically, GR genes of *L. sifanica* showed a conserved motif‐1 structure, demonstrating structural conservation similar to that observed in other coleopteran GRs. In the phylogenetic tree, they cluster on the same branch as the corresponding GRs of other beetles, forming clades consistent with functional types based on detectable pheromones. *Lsif_GR2a* and *Lsif_GR 24* cluster within the carbon dioxide receptor clade, and their positions on the phylogenetic tree are consistent with previous studies in other insects. These genes may be involved in CO_2_ detection and signal transduction (Wu et al. 2020). *Lsif_GR12a, Lsif_GR64f, Lsif_GR64,7*, and *Lsif_GR* 12 clustered with the sugar receptors of *H. cichorii* and *H., phaleratus;* they may be involved in host plant selection. However, the specific functions of these GRs require further experimental validation. *H. cichorii* and *H. phaleratus* show a pronounced GR expansion in the phylogenetic tree, which is rarely observed in other beetle species. The number of GRs in *L. sifanica* is lower than in *H. cichorii* (102) and *H. phaleratus* (86) (Wu et al. 2020), potentially because antennae are not major gustatory organs for GR gene expression. This is supported by our observation that GRs are specifically expressed in the mouthparts of *L. sifanica*. Most GR genes of *L. sifanica* are abundantly expressed in the mouthparts, which is consistent with their roles in taste recognition (Wu, Li, and Chen 2018). Interestingly, *Lsif_GR127* and *Lsif_GR7* are mainly expressed in the antennae, whereas *Lsif_GR12* is mainly expressed in the foreleg tarsus. These GRs may serve as valuable targets for investigating taste recognition specificity in *L. sifanica*. To date, no reports have described GR genes specifically expressed in the foreleg tarsus of blister beetles (Chen et al. 2014).

In insects, IRs are ligand‐gated ion channels that mediate the majority of excitatory neurotransmission (Wu et al. 2020). Similar to other beetles with expanded IR repertoires, a total of thirteen IR members in *L. sifanica* were found in this study. This is significantly reduced from the numbers identified in *H.cichorii* (50 IRs), *H. phaleratus* (45 IRs) (Wu et al. 2020), but greater than those in beetles *Plagiodera versicolora* (7 IRs) (Zhang et al. 2021) and *D. valens* (3 IRs) (Gu et al. 2015). IRs are more highly conserved in insects than ORs and GRs (Croset et al. 2010). They can be divided into divergent, species‐specific IRs and conserved antennal IRs (Benton et al. 2009). All the IR members identified in *L. sifanica* here have orthologs in other Coleoptera and can be divided into different subgroups with strong bootstrap support in phylogenetic analysis. Similar to ORco, certain IR members (IR8a and IR25a) function as conserved co‐receptors in beetles, evidenced by their consistent co‐expression with other IRs (Zhao et al. 2020). *Lsif_IR6* and *Lsif_IR25a* may belong to the co‐receptor IRs in *L. sifanica*, as they cluster with the clade containing IR8a and IR25a rather than with other IR members in the phylogenetic tree. To investigate their expression patterns, we conducted a tissue expression analysis and found most of the IRs in *L. sifanica* are highly expressed in mouthparts, consistent with previous reports (Wang et al. 2023). Notably, *Lsif_IR2a* displayed antennae‐specific expression, a pattern that is conserved across insect orders and functions in insect olfaction. *Lsif_IR56e* and *Lsif_IR 25a* are also expressed in the head. This diverges significantly from the reports on IR56e and IR25a expression in other coleopteran species. This difference is likely due to variations in olfactory receptor repertoires among distantly related species or to different evolutionary pressures. The remaining *Lsif_IRs* are expressed in multiple tissues. These divergent IRs likely participate in taste perception and food evaluation (Croset et al. 2010). The expression level of IR genes in the antennae was relatively low compared with other olfactory genes, and some IR genes may not have been detected by electrophoresis. It is unexpected that more than one gene clusters in clade A. A possible explanation is that these fragments represent portions of a single large IR gene, potentially resulting from duplication of a single IR gene in *L. sifanica*.

Insect odorant receptors (ORs) are predominantly expressed in olfactory sensory neurons and play pivotal roles in olfactory signal transduction (Wicher 2018). The olfactory process initiates when odorant molecules interact with these receptors in the sensory neurons, triggering neural signal transduction (Dobritsa et al. 2003). ORs determine the sensitivity and specificity of odorant detection and serve as the central components of the peripheral olfactory system (Xia et al. 2008). Therefore, OR proteins are highly diverse and often reflect the ecological niches of different species (Cheema et al. 2021). In the antennae of *L. sifanica*, we identified a total of 18 OR members, a number lower than that reported in *H. Cichorii* (149) and *H. phaleratus* (89) (Wu et al. 2020), but greater than in *Galeruca daurica* (10 ORs) (Li et al. 2017). Previous studies have identified 1–2 ORco genes in other Coleoptera species (Gonzalez et al. 2021; Tanaka et al. 2022), all of which clustered at the basal position of the phylogenetic tree—a finding that is consistent with our results in *L. sifanica*. *Lsif_ORs* were on the same branch and highly homologous with ORco of other species. This was consistent with fact that ORco genes are highly conserved in insect. Motif analysis showed that motif 1and motif 2 in coleopteran ORs exhibited high similarity in these genes, indicating that they may be perform the same function as *Hcic_ORco*, such as could help other *Lsif_*ORs localize to the dendritic membrane or better associate with odor molecules (Ma et al. 2019; Wu et al. 2020). Same as other beetle, we found one ORco in *L. sifanica* and showed different tissue expression. Phylogenetic analysis revealed that *Lsif_OR*co were on the same branch and were highly homologous with ORco of *H. Cichori* and other species, a pattern consistent with previous reports in other insect taxa (Zhang et al. 2024). Some OR genes might be expressed only during the larval stage (Engsontia et al. 2008) or the non‐sensory tissues (Zhang et al. 2024). Differences in the number and tissue‐specific expression patterns of ORs may reflect species‐specific chemical ecology. Some OR members were only expressed in foreleg tarsus consistent with the expression patterns of other beetles' ORs. Because some of the OR genes might be expressed only in the larva (Engsontia et al. 2008) or the non‐sensory tissues (Gong et al. 2007). The difference in numbers and expressed locations of ORs may be due to the different chemical ecology for these species. Interestingly, the *Lsif_ORco* transcript exhibited predominant expression in the foreleg tarsus, while its splice variant *Lsif_ORco_X1* showed a relatively broad expression pattern across multiple tissues. Such variation in expression profiles may be attributed to tissue‐specific alternative splicing, which is known to enhance gene functional diversity in insects. The distinct expression patterns of *Lsif_ORco* and *Lsif_ORco_X1* imply that these isoforms may perform divergent physiological roles—*Lsif_ORco* possibly functioning in specialized olfactory perception, whereas *Lsif_ORco_X1* might be involved in general sensory modulation or other regulatory processes.

SNMPs are transmembrane proteins with multiple domains, primarily involved in recognizing and transporting lipophilic odor molecules (Vogt et al. 2009). SNMP genes are expressed in the dendrite membrane of olfactory receptor neurons and play crucial roles in pheromone recognition (Forstner et al. 2008). Most studies have reported two SNMP genes in insects, namely SNMP1 and SNMP2. However, these genes appear to be missing or rare in many beetle species (Cassau and Krieger 2021). Nevertheless, multiple SNMPs have been identified in a variety of Coleopteran species (Wang et al. 2023). Five SNMPs were identified in the antennal transcriptome of *L. sifanica*, which is greater than the three SNMPs reported in *D. ponderosae* and *I. typographus* (Andersson et al. 2013). To explore potential intragenomic duplication, phylogenetic analysis revealed that each member of SNMPs clustered branches with their homologs of various beetle species rather than two close blister beetles, the reason for which may be that orthologs of *Lsif_*SNMPs are lacking in other beetles *H. Cichorii* and *H. phaleratus* . Gene replications may have appeared inversely in intraspecies of the above beetles. Tissue‐specific expression analysis showed that *Lsif_*SNMP4 and *Lsif_*SNMP2 exhibited antennae‐specific expression, although they belong to different phylogenetic branches. Conversely, *Lsif_*SNMP5 and *Lsif_*SNMP3 were dominantly expressed in abdomen skin or wings in *L. sifanica*, all of which indicated that different members may be important in different chemoreception functions for *L. sifanica*.

In summary, our transcriptomic analysis provides the first identification of numerous olfactory‐related genes in *L. sifanica*. Phylogenetic and sequence analyses revealed that each gene family exhibits characteristic features consistent with Meloidae beetles or Coleoptera in general. Our comparative analysis between *L. sifanica* and other Coleoptera species revealed significant differences in the number and expression patterns of olfactory‐related genes, reflecting species‐specific ecological adaptations. For instance, the 70 identified related proteins in this species were less than the closely related beetle species *H. cichorii* (149 ORs, 102 GRs, 50 IRs) and *H. phaleratus* (89 ORs, 86 GRs, 45 IRs), but are similar to beetle *I. typographus* (15 OBPs, 6 CSPs, 3 SNMPs, 7 IRs, 43 ORs) (Andersson et al. 2013). This suggests potential gene family expansions associated with its phytophagous lifestyle and chemical communication. Such interspecific variations among beetles highlight the evolutionary plasticity of the chemosensory system in response to ecological pressures and host adaptation. Two potential factors may account for this discrepancy, First, the dataset was derived from transcriptomes rather than genome data for their closely related species *H*. *cichorii* and *H. phaleratus* (Wu, Li, and Chen 2018). Second, the genes specifically expressed in other chemosensory organs were not found in our antennal transcriptome analyses. Additionally, the absence of larva's antennal may induce the integrality of mined family genes in adult *L. sifanica*, due to(Bajsa et al. 2015) differential expression that may exist between developmental stages in insect olfactory organs (Cao et al. 2014).

Several critical research gaps require further investigation. Most notably, the OR genes were significantly reduced compared to other Meloidae species (Capinera et al. 1985). Whether this reduction is due to gene family contraction or incomplete transcriptome assembly remains unclear. ORs generally have relatively low expression levels (Li et al. 2022), which still provide additional evidence supporting this observation. Some genes were incompletely annotated, and some novel genes could not be matched to sequences in the database used during the assembly process (Arvind et al. 2020). The exact nature of ORco types remains to be elucidated (Zhang et al. 2021). Future studies should investigate whether the related candidates form heterodimers with other receptors like GRs or with each other to build functional receptors. Additionally, their potential channel functions in non‐olfactory processes warrant further exploration (Christensen et al. 1988). Previous studies have reported that some olfactory‐related genes exhibit higher expression levels in non‐olfactory tissues than in the antennae. These members are highly susceptible to omission in the antennal transcriptome analysis. In this study, we analyzed only the expression patterns of olfactory genes in adult *L. sifanica* without conducting functional characterization. Therefore, the roles of these genes in olfactory processes remain unclear. Future studies will employ approaches such as olfactory receptor localization, heterologous protein expression, and RNA interference to investigate the functions of *L. sifanica* olfactory genes in odor recognition and other physiological processes (Field et al. 2000). In this experiment, only RT‐PCR analysis of the specific expression of different olfactory genes in different tissues was done, and no expression differences between male and female were made. Furthermore, functional characterization of these olfactory genes remains to be conducted (Xing et al. 2021). Cantharidin (C_10_H_12_O_4_, CTD) (Capinera et al. 1985) synthesized in vivo is a complex physiological process controlled by a variety of factors. However, relevant olfactory genes were not identified in this study. Future work should focus on identifying these genes and elucidating their potential roles in the biosynthesis and detection of cantharidin.

## Author Contributions

**Feng Zhou:** investigation (equal), project administration (equal), supervision (equal). **Zhuan‐xia Li:** data curation (equal), formal analysis (equal), methodology (equal), resources (equal), software (equal), writing – original draft (equal), writing – review and editing (equal). **Jia‐ni Chen:** conceptualization (equal), methodology (equal), resources (equal), software (equal). **Xin‐ge Song:** conceptualization (equal), data curation (equal), investigation (equal). **Shu‐ning Sun:** conceptualization (equal), software (equal), supervision (equal). **Yu‐ying Zhang:** validation (equal), visualization (equal). **Li‐yuan Yao:** conceptualization (equal), investigation (equal), resources (equal). **Yu‐qin Wang:** supervision (equal), validation (equal). **Xin‐yu Sun:** resources (equal). **Li‐xia Wan:** investigation (equal).

## Funding

This study was supported by the National Natural Science Foundation of China (No. 32160127 and No. 32360670) and the Science and Technology Projects of Xizang Autonomous Region, China (Grant No. XZ202501ZY0018).

## Conflicts of Interest

The authors declare no conflicts of interest.

## Supporting information


**Appendix S1:** ece372634‐sup‐0001‐AppendixS1.docx.

**Appendix S2:** ece372634‐sup‐0002‐AppendixS2.zip.

## Data Availability

The genome sequence data that support the findings of this study are openly available in NCBI at https://www.ncbi.nlm.nih.gov. The associated BioProject, SRA, and BioSample numbers are PRJNA1221373, SRR32307458, SRR32307457, SRR32307459 and SAMN46739937, respectively.
