# Supplementary material for: Chemosensory System Decoding: Transcriptome‐Wide Identification and Expression Profiling of Olfactory Genes in Lytta sifanica
Source: Ecol Evol. 2025 Dec 10;15(12):e72634. doi: 10.1002/ece3.72634 (PMC12690220; doi:10.1002/ece3.72634)
Supplement: Supplementary file 1 — Appendix S1: ece372634‐sup‐0001‐AppendixS1.docx. [file ECE3-15-e72634-s002.docx]

**Supplementary information**


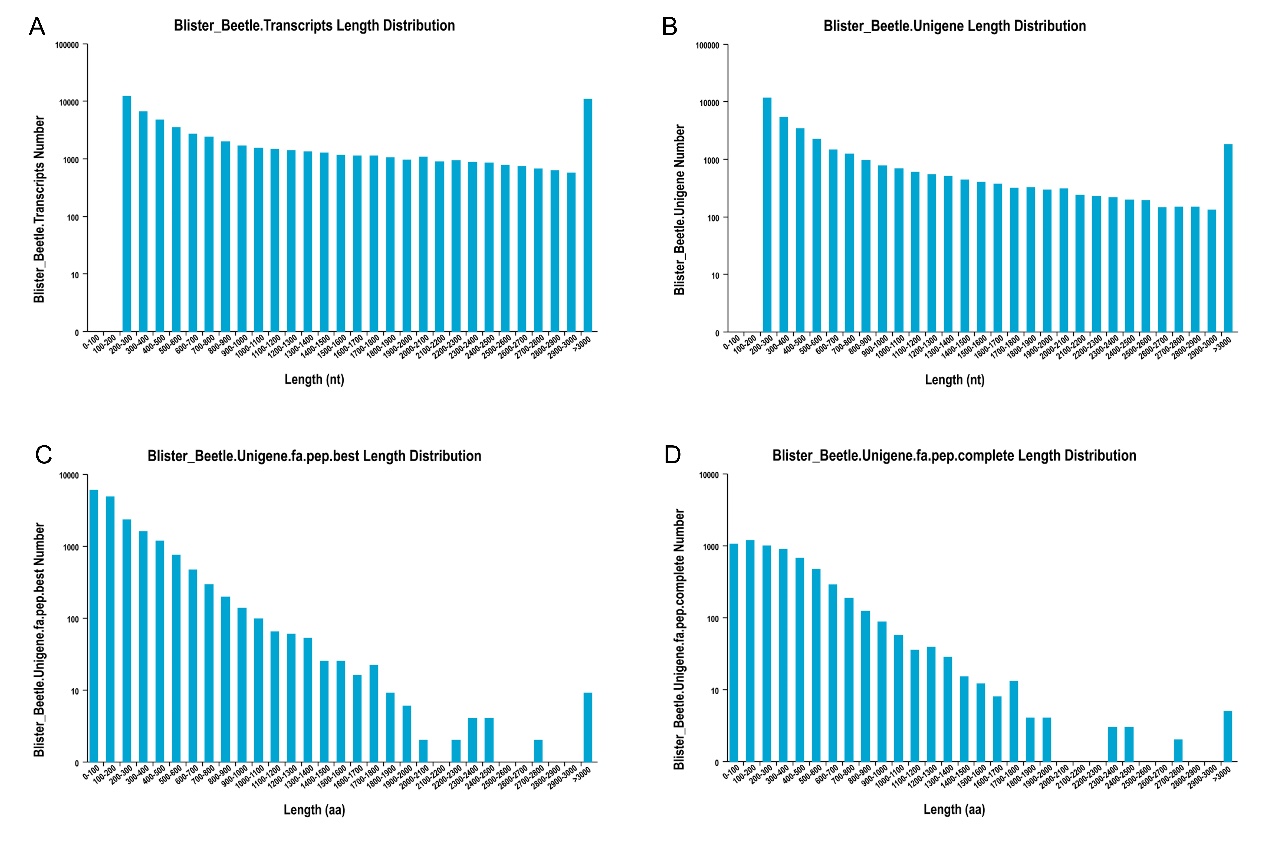


**Supplementary Figure S1.** **The Length distribution of Unigenes and Transcripts+Unigene structural analysis, including CDS prediction.** (A) The Length distribution of Transcripts of *Lytta sifanica*. X-axis is length range of Transcripts. Y-axis is the number of Transcripts fall into the range. (B) The Length distribution of Unigene of *Lytta sifanica.* X-axis is length range of Unigene. Y-axis is the number of Unigene fall into the range. (C) Length distribution of amino acid sequence of predicted orfs. Y-axis is the number of suquences predicted to the complete coding region. X-axis is length range of sequences. (D) Length distribution of amino acid sequence of predicted orfs. Y-axis is the number of suquences predicted to the complete coding region. X-axis is length range of sequences.


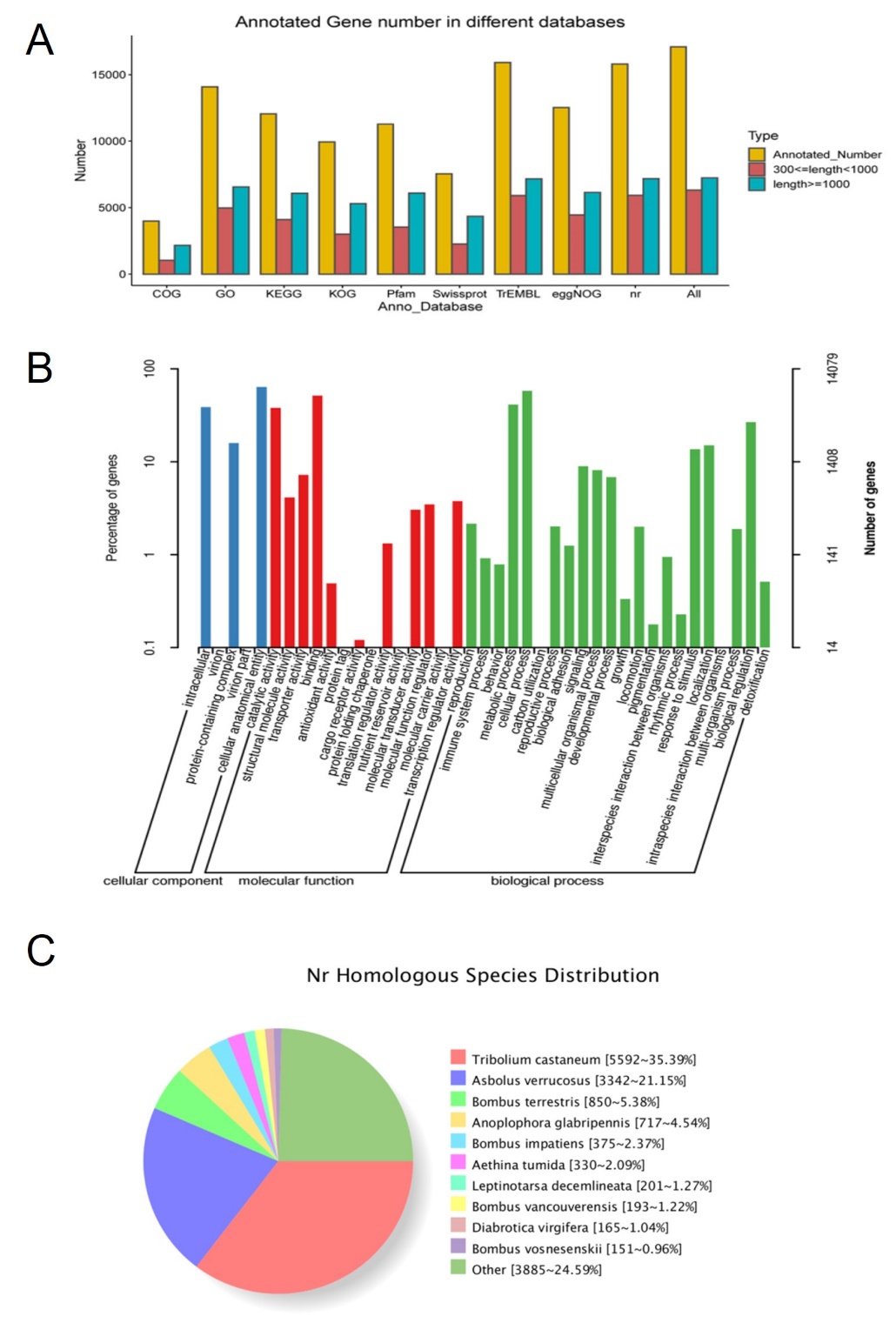


**Supplementary Figure S2. Contents of statistical figures on annotations in each database.** (A) Annotated Gene number in different databases. The horizontal coordinates of diagram represent Annotated database and longitudinal coordinate represent number, Annotated number in orange, 300<=length<1000 in red, length=>1000 in blue. (B) Gene Ontology analysis of genes in the Illumina de novo transcriptome of *L. sifanica*. The results are summarized into three categories: cellular component molecular function and biological process. Y-axis on the left is the percentage of the genes annotated to this term in total number of annotated genes. The Y-axis on the right is the number of genes annotated to the term. (C) The Nr Homologous Species Distribution. Species distribution as a percentage of the total homologous sequences with an E-value >=1.0E^-5^.


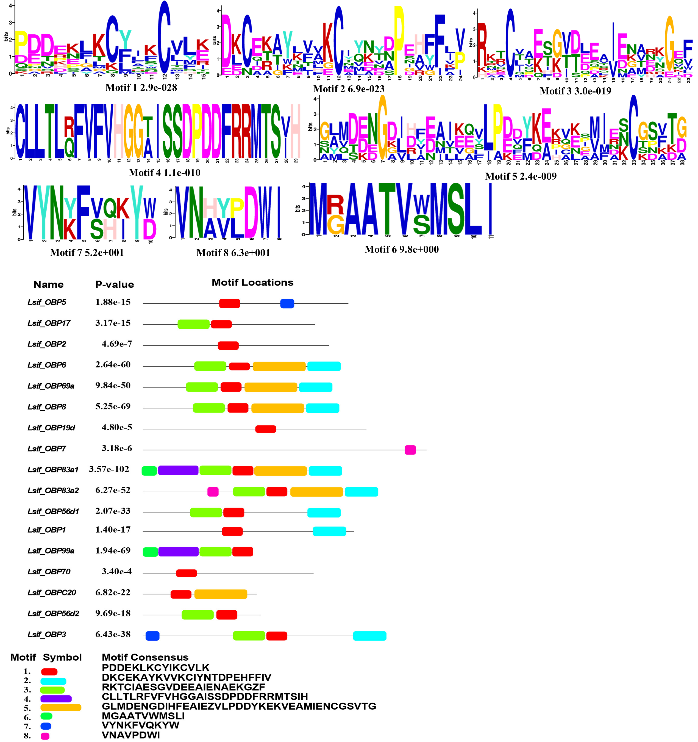


**Supplementary Figure S3. The motif analysis of OBPs.** Motif analysis of *L. sifanica* OBPs, Parameters used for motif discovery were: minimum width = 6, maximum width = 10, maximum number of motifs to find = 8. The upper parts listed the eight motifs discovered in the *L. sifanica* OBPs. The lower parts indicate approximate locations of each motif on the protein sequence. The numbers in the boxes correspond to the numbered motifs in the upper part of the figure, where the small number represents high conservation, each motif on the protein sequences starting from the N-terminal to C-terminal.

**
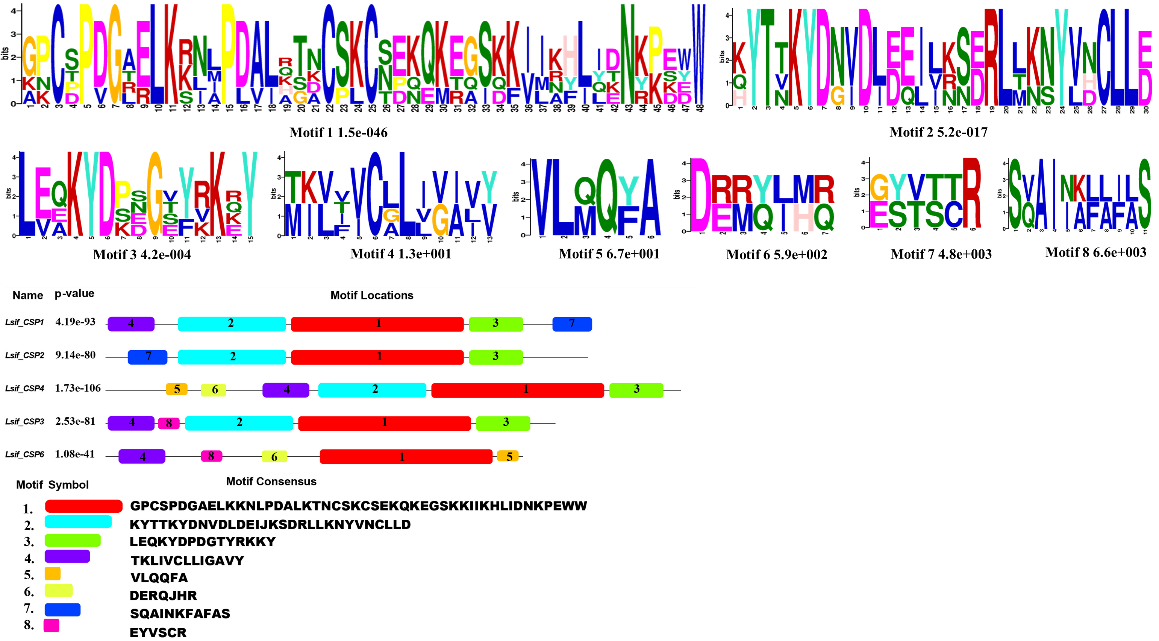
**

**Supplementary Figure S4. The motif analysis of CSPs.**

**
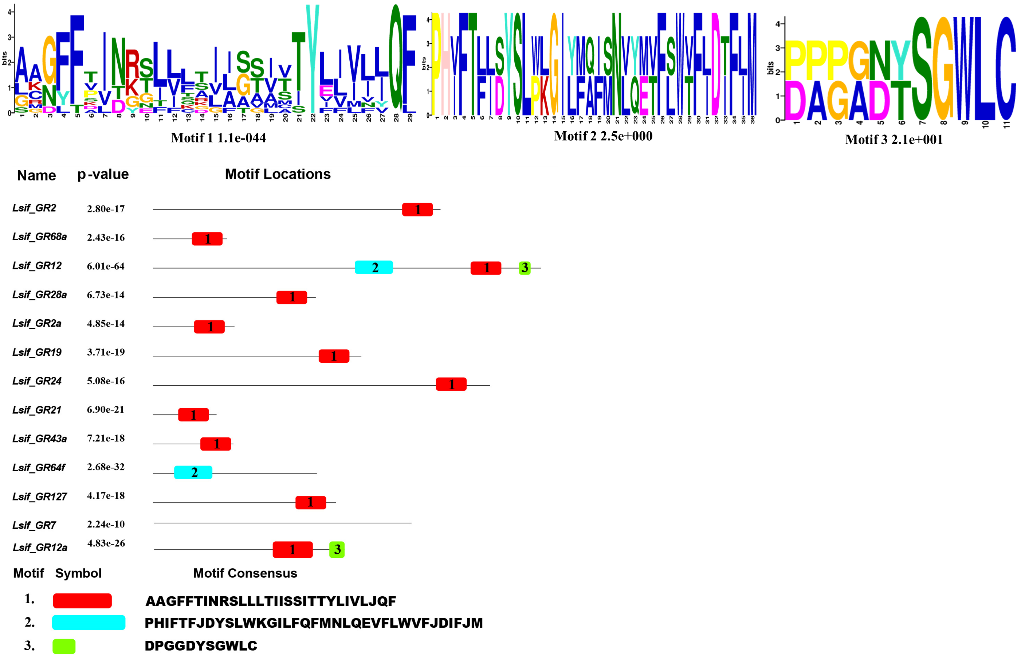
**

**Supplementary Figure S5. The motif analysis of GRs.**


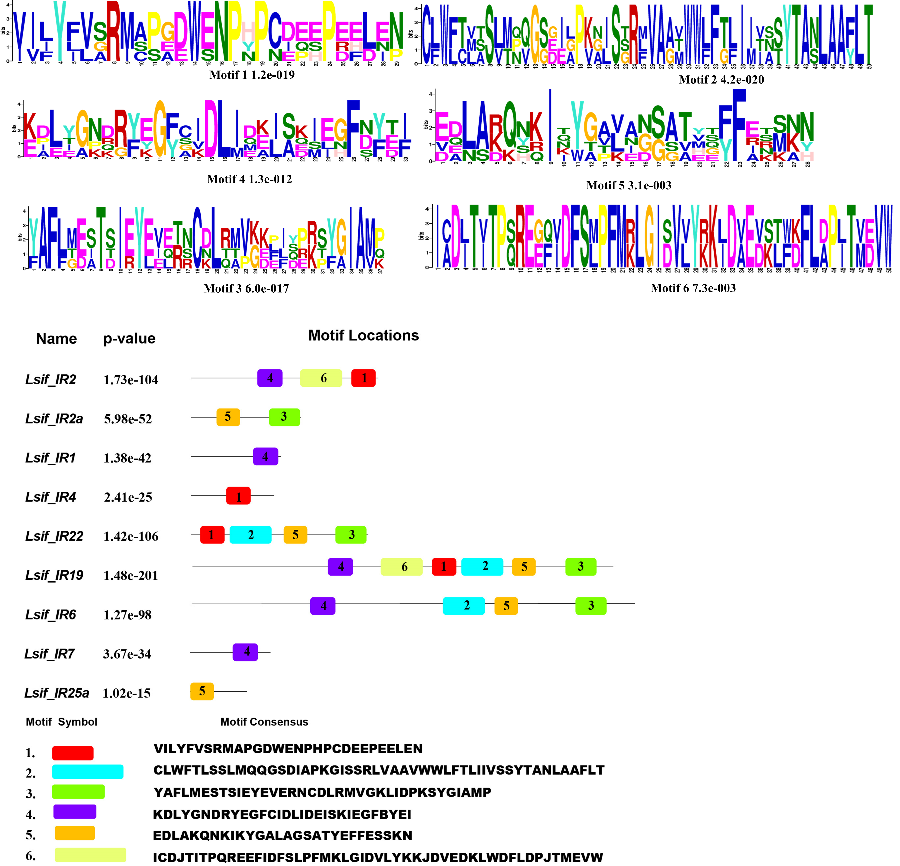


**Supplementary Figure S6. The motif analysis of IRs.**

**
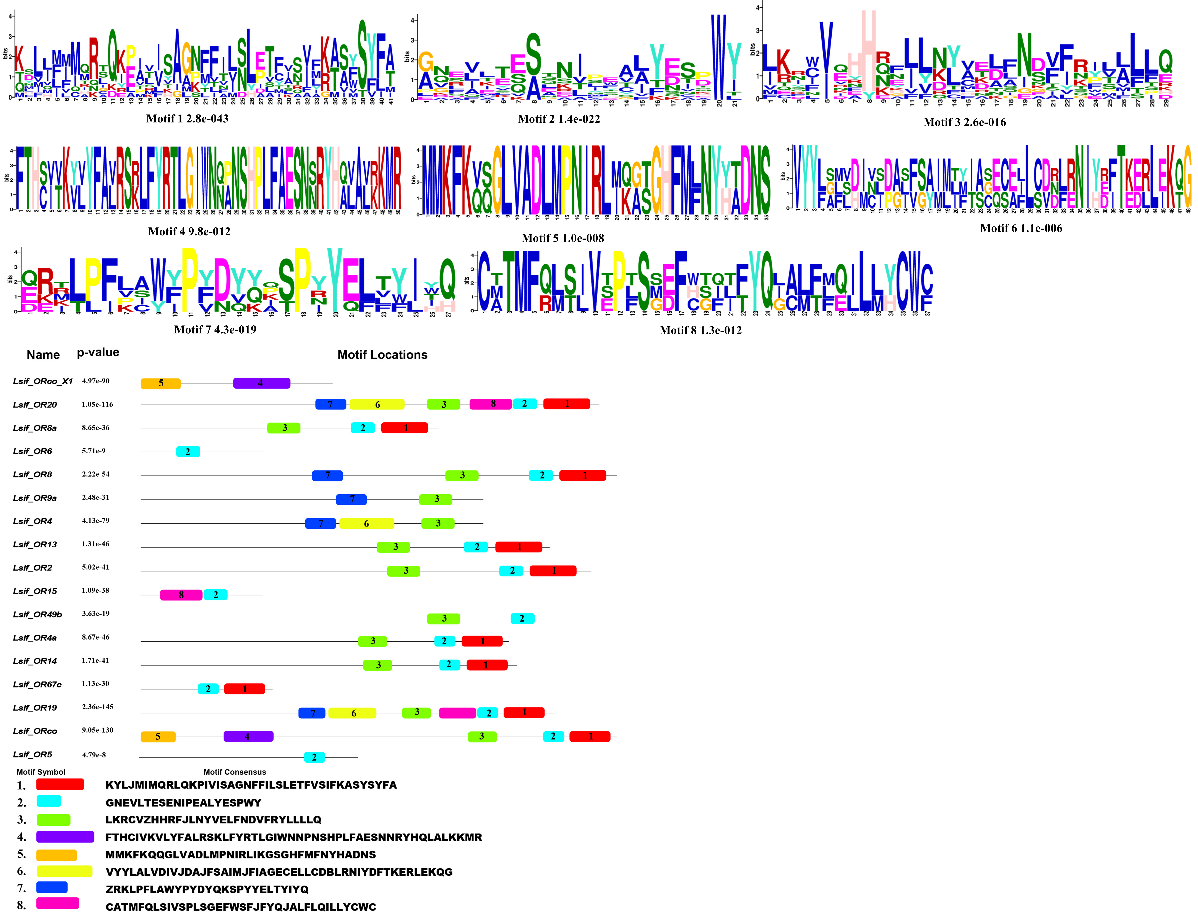
**

**Supplementary Figure S7. The motif analysis of ORs.**


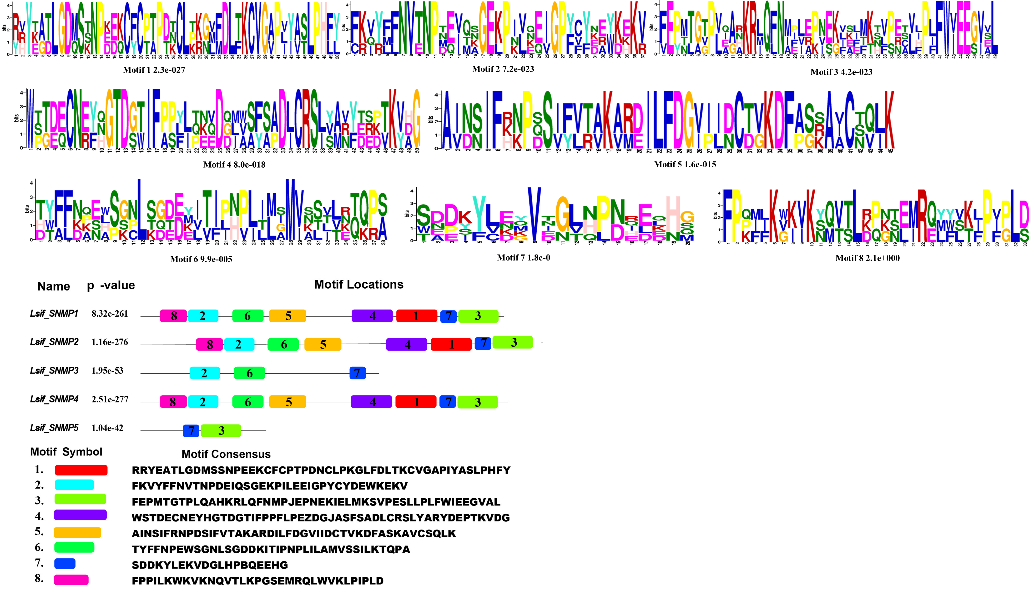


**Supplementary Figure S8. The motif analysis of SNMPs.**


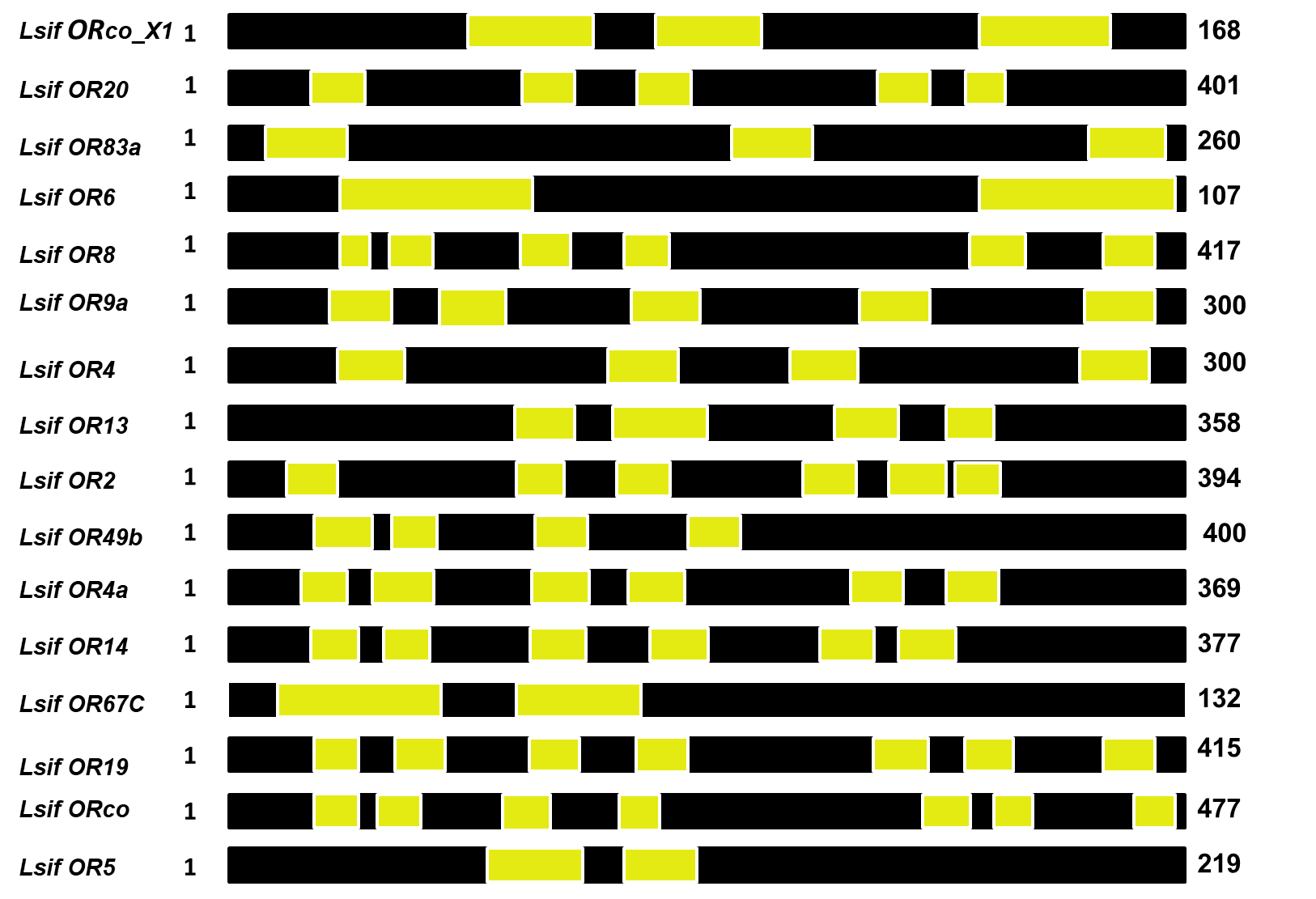


**Supplementary Figure S9. The prediction of transmembrane structure of ORs.**


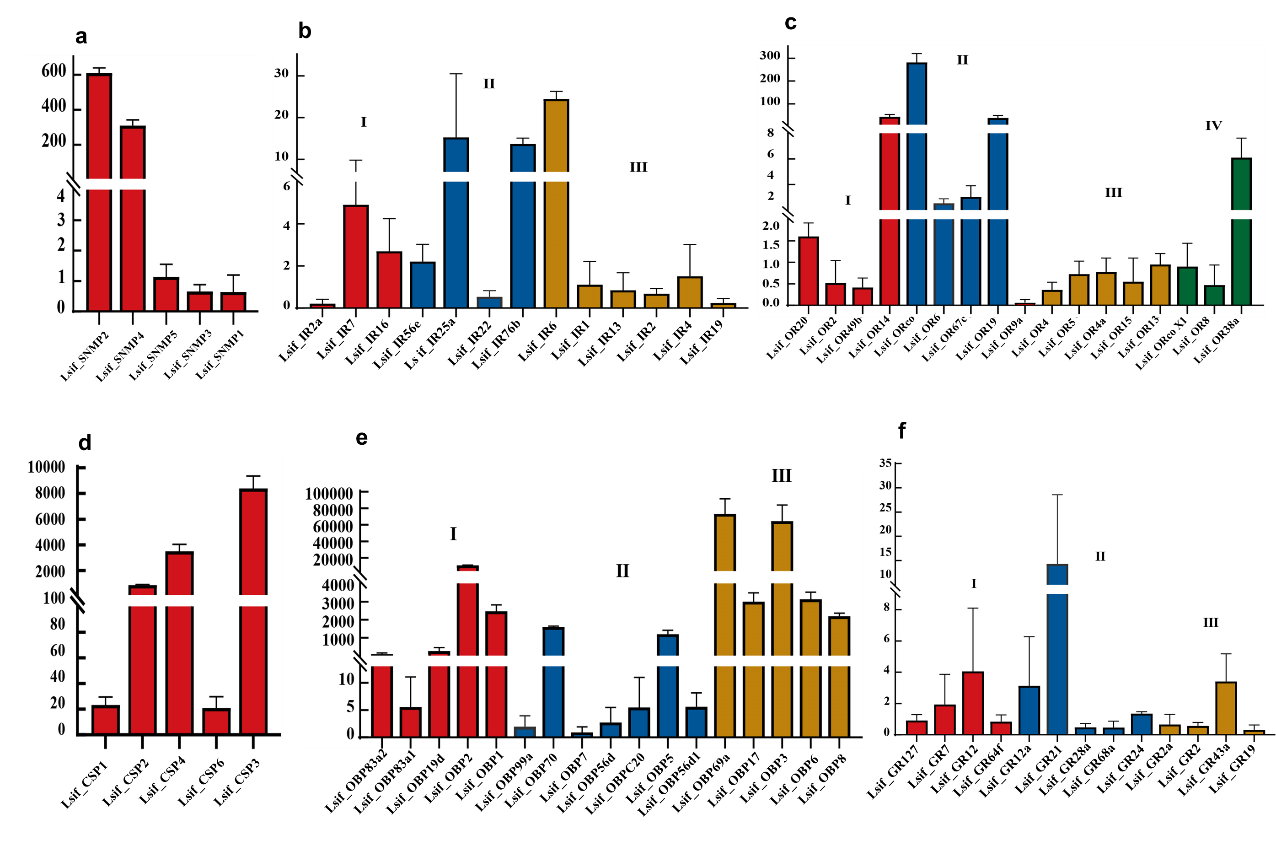


**Supplementary Figure S10. The expression level of olfactory related proteins.** FPKM value histogram different genes. (a) sensory neuron membrane proteins (SNMPs), (b) ionotropic receptors (IRs) (c) odorant receptors (ORs), (d) chemosensory proteins (CSPs), (e) odorant binding proteins (OBPs), (f) gustatory receptors (GRs)

TableS1. Candidate chemosensory genes of *Lytta sifanica* antennal transcriptome

TableS2. Total number of olfactory related genes from eleven Species

TableS3.Amino acid sequences of other insect used in the phylogenetic analyses

TableS4. Part of the annotation information of the *Lytta sifanica* sequences

TableS5. Primer pairs used for RT-PCR
